# Cross-Kingdom Pathogenesis of *Pantoea alfalfae* CQ10: Insights from Transcriptome and Proteome Analyses

**DOI:** 10.3390/microorganisms12112197

**Published:** 2024-10-30

**Authors:** Jing Su, Bo Yao, Rong Huang, Xiaoni Liu, Zhenfen Zhang, Yong Zhang

**Affiliations:** 1 College of Veterinary Medicine, Gansu Agricultural University, Lanzhou 730070, China; 107332114011@st.gsau.edu.cn; 2 Key Laboratory of Grassland Ecosystem, Ministry of Education, Pratacultural College, Gansu Agricultural University, Lanzhou 730070, China; yaobo@st.gsau.edu.cn (B.Y.); huangrong@st.gsau.edu.cn (R.H.); liuxn@gsau.edu.cn (X.L.)

**Keywords:** plant and animal cross-kingdom bacteria, grassland agroecosystems, dual RNA-seq, proteome sequencing, virulence gene, pathological tissue sections

## Abstract

In grassland agroecosystems, some plant pathogenic bacteria can cause disease in animals. These strains are known as plant and animal cross-kingdom pathogenic bacteria. In this study, we established an alfalfa root infection model and a mouse model via the gavage administration of the *Pantoea alfalfae* CQ10 (CQ10) bacterial suspension. It was confirmed that the CQ10 strain caused bacterial leaf blight of alfalfa. Mice inoculated with 0.4 mL of 10^9^ cfu/mL bacterial suspension developed clinical symptoms 48 h later, such as diminished vitality, tendencies to huddle, and lack of appetite, including severe lesions in stomach, liver, kidney, and spleen tissues. CQ10 strains were isolated from mouse feces at different time points of inoculation. Thus, CQ10 is a plant and animal cross-kingdom pathogenic bacterium. Transcriptome and proteome analyses showed that biofilm and iron uptake are important virulence factors of the pathogen CQ10, among which *Bap* and *Lpp* regulating biofilm are the key cross-kingdom virulence genes of CQ10. From an evolutionary perspective, insights gained from this dual animal–plant pathogen system may help to elucidate the molecular basis underlying the host specificity of bacterial pathogens. The result provides a theoretical basis for the risk assessment, prevention, and control strategies of new pathogenic bacteria entering a new region.

## 1. Introduction

Human health and wellbeing are not isolated but are inextricably linked to the health of other ecosystem components, such as soil, plants, and animals [1]. The health of ecosystems relies heavily on the contribution of microbial communities, which are interconnected with different organisms and form a circular loop [2]. The health of grassland agroecosystems relies on the contribution of grass, soil, livestock, and human microbial communities and are linked to each other through dispersal. Animal production is one of core productivities and identifying factors in grassland agroecosystems, which is one of the criteria of agricultural modernization as well [3]. Recently, climate change (such as changes to temperature and moisture) and the intensification of grazing and animal farming have caused an increase in the prevalence of diseases affecting plants and animals [4,5,6]. At the same time, some plant pathogens not only cause serious losses in quality and yield of forage but also produce toxins that can cause symptoms, such as digestive system disturbances or intoxication in mammalian hosts, including livestock and humans [7]. Recent studies have also presented some evidence of pathogenic bacteria causing diseases in both plant and animal hosts. It was reported that *Pseudomonas aeruginosa* can not only cause soft rot in *Arabidopsis thaliana* but also can lead to infection and death in mice (*Mus musculu*) [8]. The *Erwinia persicina* strain Cp2 was isolated from the internal tissue of alfalfa seeds, which have been shown to cause necrosis or wilting in more than 20 species of legume forage crops, such as alfalfa (*Medicago sativa*) and soybean (*Glycine max*) [9,10]. Meanwhile, Cp2 strains can provoke several symptoms, including rumina flatulence, congested eye, lymphatic inflammation, and other symptoms in *Ovis aries* [11]. The strain can also provoke other problems, such as hepatic necrosis, disseminated intravascular coagulation of kidneys tissues, and other symptoms in mice [12]. Furthermore, some pathogenic bacteria belonging to the *Pantoea* can bring about cross-kingdom infection in plants and animals. For instance, *Pantoea agglomerans* can cause pathogens in plants, such as pea (*Pisum sativum*) and wheat (*Triticum aestivum*) [13], as well as infecting humans and leading to bacteremia and septicemia [14]. In our laboratory, the pathogenicity of *P. alfalfae* CQ10 (CQ10) of alfalfa seed-borne bacteria on alfalfa was investigated in the previous work [9], but it is not clear whether the bacterium is pathogenic to animal hosts.

If CQ10 strains also have the potential to infect both plants and animals in grassland agroecosystems, the strains can easily enter the bodies of animals through feeding. In the event of widespread infestation, there will be incalculable losses to forage quality, livestock, and grassland development. Hence, clarifying the pathogenicity of CQ10 to animals is crucial in understanding the infection risks of the bacterium in the grassland agroecosystems. These results will offer a theoretical basis for the risk assessment, prevention, and control strategies of new pathogenic bacteria entering a new region. At the same time, in the global climate change and global trade context, there is the potential for cross-kingdom research into pathogenic bacteria of risk that can alert decision makers, from farm managers to national quarantine systems and biosecurity sectors, to the likely need for action and provide decision support for targeting responses. Given such a “dual” animal–plant pathogen, it would be interesting from an evolutionary perspective to determine which, if any, bacterial virulence factors are involved in both plant and animal pathogenesis. Pathogenic bacteria use a range of virulence strategies to infect their hosts, and the virulence factors and virulence proteins involved in this process are diverse and complex. More recently, transcriptomic and proteomic approaches have proved to be excellent tools to characterize and understand the dynamic interplay between host and pathogen [15,16]. Due to the incomplete agreement between biological events described at the gene level and the protein level, the combination of the transcriptome and proteome is advantageous and extensively applied, as it allows researchers to obtain a comprehensive map of physiological changes in organisms and elucidate the physiological regulatory mechanisms of organisms from different biological perspectives [17,18]. Here, this study employs dual RNA-seq and proteomics technologies, along with bioinformatics methods, to investigate the key virulence factors linked to CQ10 infection and the critical disease resistance of genes/proteins that alfalfa and mice respond to. It will provide new targets and optimal entry points for studying the virulence attributes of CQ10 in plant and animal cross-kingdom infection and also provides mechanistic insights into the interaction network between animal and plant hosts and cross-kingdom pathogenic bacteria. In addition, insights gained from this dual animal–plant pathogen system may contribute to the prevention and control of the invasion and spread of cross-kingdom pathogenic bacteria in grassland agroecosystems.

## 2. Materials and Methods

### 2.1. Bacterial Strains

CQ10 strains were isolated from the internal tissue of alfalfa seeds and stored at −80 °C [9]. The CQ10 strain was provided by the *Laboratory of Pasture Pathology, Pratacultural College*, Gansu Agricultural University, China. First, the activated bacterial strains were streaked for inoculum on nutrient agar media (NA: 3 g beef extract, 10 g peptone, 5 g NaCl, 18 g agar) at 28 °C. Next, strains were inoculated in NB (NB: 3 g beef extract, 10 g peptone, 5 g NaCl) for 36 h at 28 °C. After precipitation and centrifugation for 10 min at 12.000× *g*, the pellet was washed with sterile water and the bacterial suspension was diluted in sterile water to 10^9^ cfu/mL. According to the bacterial growth curve of CQ10, an incubation time of 36 h has been suggested as a threshold for cell growth.

### 2.2. Plant Materials

*M. sativa* “Juneng 995” was used as a test material for an alfalfa root infection model, which has been proven to exhibit pathogenic symptoms at the seedling stage after being infected with 10^9^ cfu/mL CQ10. Glass transparent growth bottles were used for plant growth experiments [19]. The strain caused bacterial leaf blight of alfalfa after inoculation from the roots after 21 days. These typical symptoms included leaf spots, chlorosis, necrosis, and leaf wilting, and the root growth of alfalfa was severely inhibited [9]. All of the methods used in this study have been previously reported by our laboratory [9]. Furthermore, all symptomatic alfalfa leaves from alfalfa root infection experiments were harvested after 21 d, frozen in liquid nitrogen, and later, stored at −80 °C for transcriptome and proteome analyses in this study.

### 2.3. Animals and Treatments

Kunming (KM) mice (specific-pathogen-free grade, 18–22 g) were purchased from the Lan Zhou Veterinary Research Institute, Chinese Academy of Agricultural Science and maintained in the Specific-Pathogen-Free (SPF)-Grade Trial Animal Center (Lanzhou university, Lanzhou, China). The animal experiments were approved by the animal ethics committee of Gansu Agricultural University (License: SCXK (Gan) 2010-0002), and the mice were fed in the SPF-Grade Trial Animal Center of Lanzhou University, China.

The bacterial inoculation experiment was divided into two treatment groups: Group CK, sterile water (control); Group CQ10. Hence, a total of 64 SPF-grade KM mice were randomly divided into two groups, Group CK and Group CQ10, with 16 males and females per group. Additionally, for eliminating the effects of interpopulation, 32 mice were randomly divided into eight groups and placed in cages (4 mice per cage). Prior to inoculation, groups of mice were kept on an ad libitum diet for 2 d to ensure that the mice were accommodated to the environment and did not exhibit any abnormalities. The inoculation test was conducted in a sterile environment in a biological safety cabinet. Doses were given by gavage using a stainless-steel ball-tipped gavage needle (a curved 8G needle) and 1 mL syringe. The mice were inoculated one at a time with 0.4 mL of the bacterial suspension. The mice underwent fasting for 4 h and water deprivation for 4 h prior to intragastric administration and were given food and drinking water 4 h after irrigation. Intragastric administration was performed twice a day for 3 consecutive days. After gavage, the growth in the mice was observed and recorded.

### 2.4. Measurement of Indices

After the end of gavage, food intake and body mass of mice were measured at 24 h, 48 h, 72 h, and 96 h after the inoculation. Food intake was measured from the difference between the amount fed and the amount remaining. Then, the blood samples of 4 mice from each of the experimental groups were collected, and the mice were sacrificed for tissue collection. The blood of all the mice was collected into a vacuum blood collection tube through an eyeball extirpating method. Blood counts were measured by Wuhan Sevier Company (Sevier Biological technology Co., Ltd., Wuhan, China). The weight of the heart, liver, spleen, lungs, kidney, and stomach of all the mice were recorded at necropsy, and relative organ weights were calculated as a percentage of body weight. Furthermore, these tissues obtained through dissection were promptly preserved in a 4% paraformaldehyde fixative solution. These preserved tissues were dispatched to the Wuhan Saiweier Company for the creation of pathological sections. All sections were scanned and analyzed with the TissueFAXS imaging system (Tissue Gnostics, Vienna, Austria).

### 2.5. Isolation and Identification of Bacteria from Mouse Feces and Tissues

In all cases, experiments were conducted on a mixture of feces from both male and female mice. Fecal pellets were collected from the mice by putting each mouse into its own cage 1 h after feeding, empty of bedding, with no access to food or water. Feces from each mouse were collected soon after defecation. Feces from mice of the same cage were put into the same 1.5 mL microcentrifuge tube. This procedure lasted no longer than one hour, after which mice were placed back into their respective cages. Then, isolation and identification of bacterial isolates from the dung was carried out by serial dilution method [20]. Each of those morphologically differentiable bacterial colonies was repeatedly streaked on plates of NA medium for purification until a complete isolation was achieved, at which point the colony was cryopreserved at −80 °C in glycerol stocks. Colonies that were purified and similar to CQ10 were analyzed for 16 S rRNA sequences and PCR amplification. The isolation of endophytic bacteria from the heart, lung, liver, stomach, kidney, and spleen was carried out in the same way as for the isolation of bacteria from feces.

### 2.6. Dual RNA-Seq Analysis

#### 2.6.1. Sample Preparation

Based on observations of the pathological sections of the organ tissues in the mice, stomach tissues exhibited the most severe lesions and slight erosion in gastric epithelium, and the gastric tissue mucosa lamina propria pathological section was messy. Thus, the samples for dual RNA-seq analysis were selected from CQ10 bacterial cells (CQ10), healthy and diseased junctions of alfalfa leaves (CQ10-P), and the pathological stomach tissues of mice (CQ10-A) in the study (Table 1). First, diseased leaves were cut into small pieces of tissue (approximately 0.5 × 0.5 cm^2^) by using a sterile knife at the interface between healthy and diseased leaves. Then, samples were then taken in 2 mL grinding tubes, crushed, and ground, and TRIzol reagent (Invitrogen, Carlsbad, CA, USA) was used to extract the total RNA from each sample. Three biological replicates were set for each sample. The RNA concentration and purity were evaluated using an Agilent 2100 bioanalyzer (Agilent Technologies, Santa Clara, CA, USA), and RNA integrity was determined via 1% agarose gel electrophoresis. After the test was qualified, the cDNA library construction and transcriptome sequencing were conducted by Novogene Co., Ltd. (Beijing, China).

#### 2.6.2. Screening of Differentially Expressed Genes

Libraries were sequenced on a NovaSeq6000 using PE150 sequencing. The raw sequencing reads were subjected to quality control and preprocessing to obtain clean reads. The high-quality clean reads were mapped to CQ10., *M. sativa*, and mice reference genomes by using Bowtie2 (http://bowtie-bio.sourceforge.net/bowtie2/index.shtml, accessed on 25 October 2022) for genome localization alignment and analysis. Next, the feature Counts function within the Subread software (https://subread.sourceforge.net/, accessed on 25 October 2022) package was employed to tally the number of clean reads covered by each gene aligned to the reference genome, ranging from the initial to the terminal regions. Based on the location information of gene alignment in the reference genome, the number of reads covered by each gene from start to end was calculated using the feature Counts tool in Subread software, and nonconforming clean reads were filtered out. The quantification of gene expression was performed using HTSeq version 0.9.1, and the FPKM (fragments per kilobase of exon model per million mapped fragments) method was used to eliminate the influence of different gene lengths and sequencing levels on the calculation of gene expression.

### 2.7. Proteome Analysis

The alfalfa leaves and stomach tissues were the same as those described in a previous section (sample preparation for transcriptome sequencing) (Table 2). The samples were removed from −80 °C storage and crushed on a liquid-nitrogen-cooled stage. Mouse stomach samples were sonicated in the urea lysis buffer containing 100 mM NH_4_HCO_3_ (pH = 8) and 8 M urea for 5 min on ice; alfalfa leaves samples were sonicated in urea lysis buffer containing 100 mM triethylammonium bicarbonate (TEAB, pH 8.5), 4% sodium dodecyl sulfate (SDS), and 10 mM DL-Dithiothreitol (DTT) for 5 min on ice. The lysates were centrifuged separately at 12,000× *g* for 15 min at 4 °C. The supernatants were reduced by 10 mM DTT for 1 h at 56 °C and, subsequently, alkylated with sufficient iodoacetamide for 1 h at room temperature in the dark. Next, samples were completely mixed with 4× volume of precooled acetone via vortexing and incubated at −20 °C for at least 2 h. After centrifugation, the pellet was resuspended, washed with cold acetone, and dissolved using a dissolution buffer, which contained 0.1 M triethylammonium bicarbonate (TEAB) and 8 M urea. All protein samples were subjected to 12% sodium dodecyl sulfate polyacrylamide gel electrophoresis (SDS-PAGE), all of which showed no visible sign of degradation.

Each sample protein was digested with trypsin [21], and then, the total proteins were treated to obtain peptides and measured. The peptide mixtures were dissolved in phase A of the liquid chromatography mobile phase and separated on an EASY-nLCTM 1200 UHPLC pump system equipped with a homemade adsorption column (15 cm × 150 µm, 1.9 µm) to separate the proteins and increase the proteome depth. Finally, the peptides were analyzed via liquid chromatography tandem mass spectrometry (LC-MS/MS) performed on an EASY-nLC system connected to a Q Exactive mass spectrometer (Q ExactiveTM HF-X) equipped with a nanoelectrospray ion source.

### 2.8. Equipment and Reagents

Various fractions of NA and NB were purchased from Beijing Solaibao Technology Co., Ltd. (Beijing, China). Electron microscope fixation liquid was purchased from Wuhan Sevier Biological technology Co., Ltd. (Wuhan, China) NH_4_HCO_3_, urea, TEAB, SDS, and DTT were purchased from Shanghai source leaf Biological Technology Co., Ltd. (Shanghai, China). The FastKing RT Kit (KR116) was purchased from Sangon Inc. (Shanghai, China). The TissueFAXS imaging system was provided by LZU 2nd Hospital. In addition, glass transparent growth bottles were provided the Forage Pathology Laboratory of Gansu Agricultural University. All bacterial inoculation experiment and gavage trials were carried out in biosafety cabinets (HR40-IIA2). The conical flask, tap water, growth bottles, and gavage needles in the test were sterilized in a high-temperature and high-pressure sterilization pot at 0.12 MPa and 121 °C for 21 min (ZEALWAY, Xiamen, China).

### 2.9. Identification and Analysis of Differentially Expressed Genes (DEGs) and Differentially Expressed Proteins (DEPs)

Statistical significance test of DEGs was performed by DESeq2 with R. Genes with absolute log2-transformed fold changes >0 were regarded as DEGs, with a threshold of *p*-value < 0.05. GO and KEGG pathway annotations were performed for all DEGs. Additionally, virulence factor analysis and pathogenic bacteria–host interaction analysis were performed for all DEGs. Among them, another strain of plant and animal cross-kingdom pathogen was selected, *Erwinia persicina* Cp2, discovered in the Forage Pathology Laboratory of Gansu Agricultural University, as a control strain for screening cross-kingdom virulence factors and pathogen–host interaction genes (data were derived from the transcriptome data of CQ10 with alfalfa and mice). A fold change >1.0 with a *p*-value < 0.05 was used as the primary criterion for DEPs [22]. GO and KEGG pathway annotations were performed for all DEPs.

### 2.10. Reverse Transcription Quantitative Polymerase Chain Reaction (RTq-PCR) Validations of DEGs

In order to validate the accuracy of expression abundance from RNA-Seq, eight important DEGs (*K6R05_RS19660*, *K6R05_RS18735*, *K6R05_RS17085*, *ssrA*, *K6R05_RS11135*, *tssB*, *rplP*, and *rpsS*) were selected and tested via RTq-PCR, and *rnpB* was used as the reference gene. The total RNA of the samples was extracted using a TRIzol^TM^ Kit (Invitrogen, A2010A0402) and reverse transcribed using a FastKing RT Kit (TIANGEN, KR116), according to the manufacturer’s protocols. All primers used for RTq-PCR analyses were designed by Primer Premier 5 software and are listed in Supplementary Table S1. RTq-PCR experiments were performed using a LightCycler^®^ *96* system (Roche, Indianapolis, IN, USA), and the reaction system was set up using the SuperReal PreMix Plus (SYBR Green) kit (TIANGEN, Co., Ltd., Beijing, China) with a 20 μL reaction. Reaction cycle conditions: pre-denaturation at 95 °C for 15 min, denaturation at 95 °C for 10 s, annealing at 60 °C for 30 s, extension at 95 °C for 15 s, 40 cycles, where the fluorescence was acquired. The RTq-PCR validation experiment included three replicates, each with three technical replicates. The relative expression of each DEG was calculated using the 2^−ΔΔCt^ method [23]. RTq-PCR was performed on a LightCycler 96 real-time PCR instrument (Roche).

### 2.11. Statistical Analysis

The data were collated and graphed using Excel 2021 and Origin 2022. The data are expressed as the mean ± the standard error of the mean from independent replicates and analyzed by SPSS 26.0. Furthermore, we performed a deep analysis based on DEGs and DEPs, including gene ontology (GO) enrichment analysis, Kyoto Encyclopedia of Genes and Genomes (KEGG) pathway enrichment analysis, cluster analysis, and virulence factors analysis and pathogenic bacteria–host interactions analysis with *p*-value < 0.05.

## 3. Results

### 3.1. Infection of Mice Challenged with CQ10 Strains

Compared with the CK group, the emergence of clinical symptoms was observed in the mice after inoculation, such as diminished vitality, tendencies to huddle, and lack of appetite, particularly around 48 h post inoculation, with signs of alleviation observed 72 h later. The heart, lung, liver, stomach, kidney, and spleen tissue homogenates were coated with NA medium for 16S rRNA sequences analysis. The results revealed that CQ10 strains were isolated from tissues, such as liver, stomach, kidney, and spleen, but not from heart and lung tissues. However, CQ10 strains were isolated from mouse feces at different times points of inoculation by sequencing the 16sRNA gene. Therefore, we inferred that if CQ10 strains enter the body of livestock, it will adversely affect their digestive system, and the excreta of the livestock will contaminate the soil.

### 3.2. Effect of CQ10 Strains on Food Intake and Body Mass of Mice

**Figure 1:**
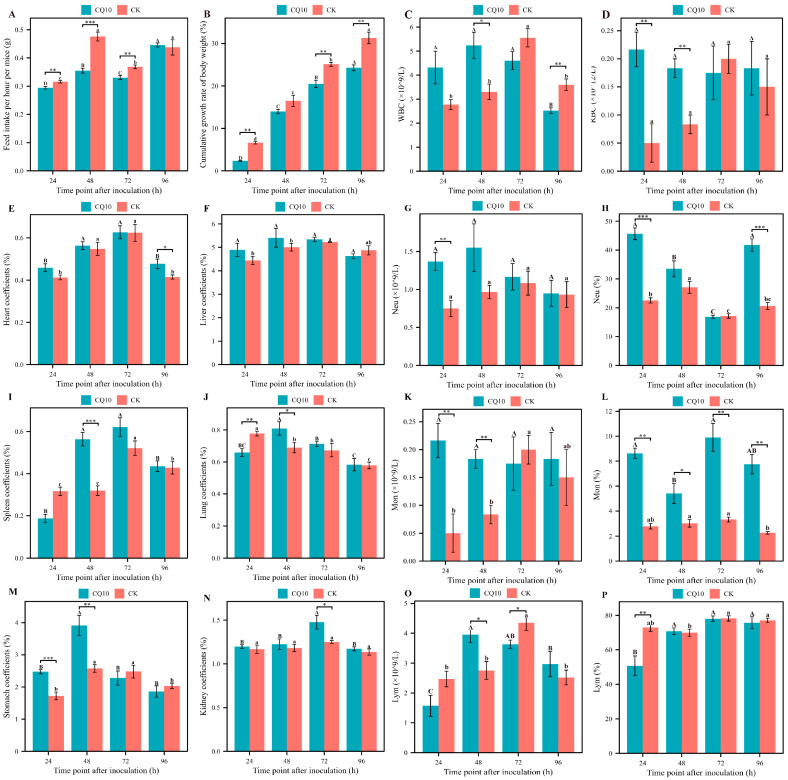
Changes in physiological and biochemical indicators of mice after inoculation. (**A**) Changes in feed intake of body weight of mice after inoculation. (**B**) Changes in cumulative growth rate of body weight of mice after inoculation. (**C**) White blood cell (WBC) counts. (**D**) Red blood cell (RBC) counts. (**E**) Heart coefficient. (**F**) Liver coefficient. (**G**) Neutrophil (Neu) counts. (**H**) Neu percentage. (**I**) Spleen coefficient. (**J**) Lung coefficient. (**K**) Monocytes (Mon) counts. (**L**) Mon percentage. (**M**) Stomach coefficient. (**N**) Kidney coefficient. (**O**) Lymphocyte (Lym) counts. (**P**) Lym percentage. Different capital letters indicate significance of differences between treatment times in the CQ10 group. Different lowercase letters indicate significance of differences between treatment times in the CK group. *, ** and *** shows the difference between the CQ10 group and CK group at the same treatment times.

The feed intake and cumulative growth rate of the body weight of mice in the CQ10 group was reduced compared with that of the mice in the CK group. The feed intake in the CQ10 group was significantly reduced by 9.38%, 28.89%, and 10.81% at 24 h, 48 h, and 72 h after inoculation (Figure 1A), and the cumulative growth rate of the body weight of mice was reduced by 65.06%, 15.12%, and 18.39%, also significantly.

### 3.3. Effect of CQ10 Strains on the Organ Coefficient of Mice

Compared with the CK group at 24 h, 48 h, and 72 h of gavage, the heart, liver, and kidney coefficients of the CQ10 group showed an increasing trend, respectively. However, only the kidney coefficients indicated a statistically significant difference at 72 h (Figure 1E,F,N). The spleen and lung coefficient were only lower than the CK group at 24 h and showed an increasing trend at other time points, but the increase was identified as statistically significant between 48 h; however, no significant difference was identified after 72 and 96 h (Figure 1I,J). Notably, CQ10 inoculation had a significant effect on the stomach coefficient, which increased significantly by 30.36% and 34.15% at 24 and 48 h and decreased at 72 h and 96 h of inoculation. However, no significant difference was observed between the 72 h and 96 h groups (Figure 1M). The results revealed that the organ coefficients of the mice showed an overall upward trend after the end of CQ10 strain suspension gavage.

### 3.4. Effect of CQ10 Strains on the Blood Routine Indexes of Mice

Figure 1 shows that the neutrophil (Neu) counts in the CQ10 group increased by 45.12% and 48.67% compared with the CK group 24 h and 48 h after gavage. At the end of 48 h, the number of Neu in the CQ10 group showed a decrease (Figure 1G). It is worth mentioning that Neu counts were, respectively, 1.88 × 10^9^/L in the CQ10 group, surpassing the upper limit of the normal range of the Neu counts (1.8 × 10^9^/L). As shown in Figure 1H, a higher percentage of Neu was found in the CQ10 group compared with the CQ10 group. The number and percentage of monocytes (Mon) of the CQ10 group was higher than the CK group during the inoculation; the number significantly increased by 76.92% and 78.26% after 24 h and 48 h (Figure 1K,L). In addition, the number of Mon had exceeded the upper limit of reference range (0.3 × 10^9^/L) after 48 h, and the percentage of Mon had exceeded the upper limit of reference range (6.0%) after 24 h, 72 h, and 96 h. Lymphocyte (Lym) counts in the CQ10 group were higher than that of the CK group after 24 h and 48 h, while the number decreased and was lower than that of the CK group after 72 h and 96 h (Figure 1O). The Lym percentage of the CQ10 group was lower than that of the CK group at each time point, but none of them was statistically significant (Figure 1P). Compared with the CK group, strain CQ10 inoculation led to an increase in the white blood cell (WBC) and red blood cell (RBC) counts after treatment for 24 h and 48 h, the WBC counts significantly increased by 38.42% after 48 h, and RBC counts significantly increased by 1.37% at 48 h (Figure 1C,D). The number of RBC and RBC counts in the CQ10 group were lower than that of the CK group after 72 h and 48 h, but none of them was statistically significant (Figure 1C,D). With the inoculation time prolonged, the platelet (PLT) counts in CQ10 group showed a decreasing trend initially, and then increased, and reached a minimum count of 7.92 × 10^11^/L after 48 h, but none of them was statistically significant (Figure S1A). The red blood cell distribution width coefficient of variation (RDW), Hb hemoglobin (HGB), hematocrit (HCT), mean cell volume (MCV), and mean corpuscular hemoglobin (MCH) concentration of mice were higher than that of the CK group, but they were not statistically significant (Figure S1B–F).

### 3.5. Effect of CQ10 Strains on the Pathological Changes of Mice

**Figure 2:**
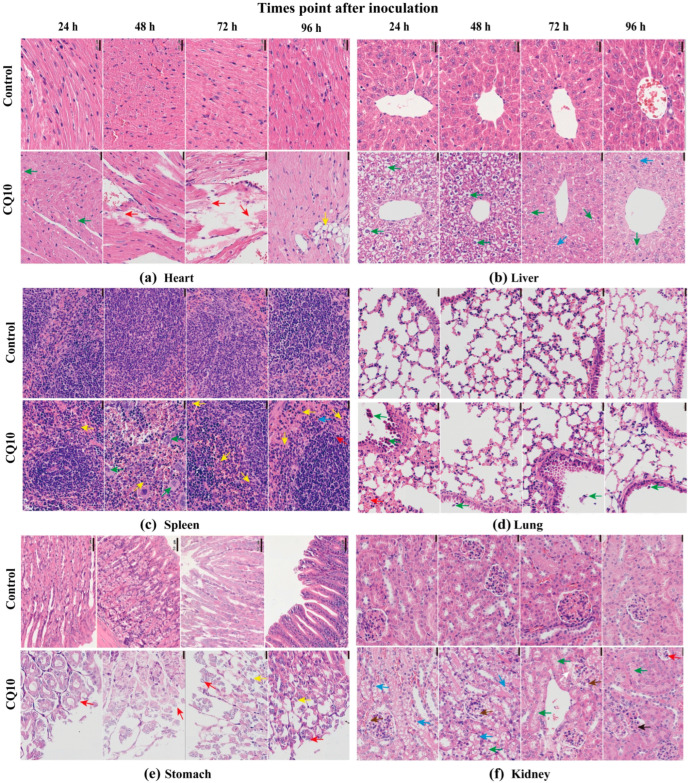
Changes in the pathological changes in mice after inoculation. (**a**) Heart. (**b**) Liver. (**c**) Spleen. (**d**) Lung. (**e**) Stomach. (**f**) Kidney.

KM mice showed minimal cardiomyocyte mild steatosis with light evidence of cytoplasmic vacuolization 24 h after gavage (green arrow) (Figure 2a). Some myocardial fibers gradually dissolved and fractured after treatment for 48 h and 72 h (red arrow) (Figure 2a). The heart tissue exhibited moderately abnormal heart tissue structure characterized by myocardial fibers showed infiltration by fat cells after 96 h (yellow arrow) (Figure 2a). The thickening of alveolar walls and mild alveolar epithelial cell hyperplasia (red arrow) accompanied by the sloughing of bronchial epithelial cells was also observed in the CQ10 group after gavage (green arrow) (Figure 2b). The liver tissue exhibited moderately abnormal liver tissue structure characterized by hepatocytes showed vacuolated cytoplasm with mild oedema (green arrow) for 24 h and 48 h after gavage (Figure 2b). The symptoms were alleviated, and liver cells exhibited the slight steatosis shown by many cytoplasmic fat vacuoles in the CQ10 group compared with the CK group after 72 h (Figure 2b). Other pathologic manifestations included mild-to-moderate spleen tissue inflammation with the infiltration of predominant inflammatory cells and few neutrophils, along with significantly increased multinuclear macrophages (green arrow) after treatment for 24 h, 48 h, and 72 h (Figure 2c). As shown in Figure 2d, small amounts of cell necrosis and hemosiderin deposition were observed in the lung at 96 h (blue arrow). In CQ10 group, the stomach tissue exhibited mildly to severely abnormal stomach tissue structure characterized by slight erosion in the gastric epithelium, the lamina propria being exposed, and the gastric tissue mucosa lamina propria pathological section being messy, as shown by the red arrow (Figure 2e). In addition, mice showed glomerular capillary congestion, swelling of the renal tubular epithelial cells, and loss of the brush border of the renal tubule, and cytosol appeared vacuolated after 24 h and 48 h (blue arrow) (Figure 2e). After 72 h, similar to the symptoms observed at 48 h, a small amount of proteinaceous material was present in the space of some glomeruli (white arrow). Besides, lobulation was observed in certain glomeruli after 96 h (black arrow) (Figure 2f). In conclusion, after oral gavage of CQ10 suspension to mice, we have shown that CQ10 has an ability to infect mice, and the most severe manifestations of pathogenicity were in gastric tissue.

### 3.6. CQ10 on Alfalfa and Mouse Cross-Kingdom Infection

Our previous study has proven that CQ10 causes alfalfa leaf blight [9]. In summary, CQ10 is a potential cross-kingdom pathogenic bacterium and has the ability to infect alfalfa and mice at the same time. In addition, samples of lesioned alfalfa leaves and mice stomach tissues infected by CQ10 were selected to carry out transcriptomic and proteomic analyses.

### 3.7. Dual RNA-Seq, DEGs Analysis, GO, and KEGG Enrichment

**Figure 3:**
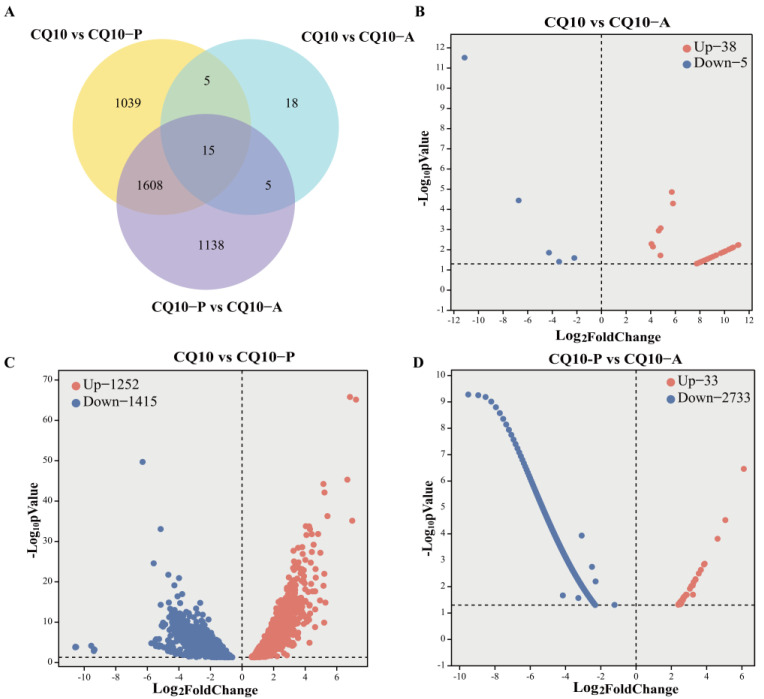
Venn analysis and the volcano plot of number of DEGs in three groups. (**A**) Venn analysis of total DEGs. (**B**) Volcano plot of number of DEGs in the CQ10 group and CQ10-A group. (**C**) Volcano plot of number of DEGs in the CQ10 group and CQ10-P group. (**D**) Volcano plot of number of DEGs in the CQ10-P group and CQ10-A group. DEGs: differently expression genes; “up” and “down” separately represent up-/downregulated expression of genes, the same as below.

The transcriptome of leaf lesions of alfalfa and histopathological stomach tissue of mice were analyzed to explore the pathogenic mechanism of CQ10. The RNA-Seq results are shown in Table S2. A total of nine samples, including three treatments and three triplicates, were transcriptome sequenced, in which 704,141,574 clean reads were filtered from 720,598,204 raw reads, including 106 Gb of clean bases. The average values of Q20 and Q30 for nine samples were 97.58% and 93.09%, respectively, which reflect the high quality of the obtained bases. After the alignment of the clean reads to the strain CQ10 reference genome, the results showed a mapping rate greater than 92% in CQ10. The mapping rate of reads expressed by strain CQ10 in alfalfa ranges between 2.55% and 3.33%, while the mapping rate of reads expressed in mice ranges between 0.04% and 0.12% (Table S2). Subsequently, the DEGs of three groups were identified and visualized. A total of 43 DEGs were found in the CQ10/CQ10-A groups, of which 38 were upregulated and 5 were downregulated. In the CQ10/CQ10-P groups, there were 2667 DEGs, of which 1252 were upregulated and 1415 were downregulated. In the CQ10-A/CQ10-P groups, there were 2766 DEGs, of which 33 were upregulated and 2733 were downregulated (Figure 3).

Furthermore, the FPKM of differential genes’ centration and normalized expressions was used to map the clustering heatmap of hierarchical cluster analysis. The clustering heatmap showed a significant difference for the expression of genes between the CQ10/CQ10-P group and CQ10/CQ10-A and CQ10-A/CQ10-P groups (Figure S2). The genes expressed by the CQ10 strain differed between alfalfa and mice. Specifically, a subset of genes in alfalfa exhibited induction through upregulation or downregulation; whereas, this subset did not elicit a similar response in mice. Conversely, another subset of genes showed induction through upregulation or downregulation in mice, but not in alfalfa. Therefore, we suggest that these genes are involved in the response to alfalfa leaf blight and stomach tissue pathological changes in mice, as well as in regulating disease resistance. The aim of this study was to identify the genes whose expressions are upregulated or downregulated in both hosts. The genes expressed by the CQ10 strain differed between alfalfa and mice, as illustrated in Figure S2. Specifically, a subset of genes in alfalfa exhibited induction through upregulation or downregulation; whereas, this subset did not elicit a similar response in mice. Conversely, another subset of genes showed induction through upregulation or downregulation in mice, but not in alfalfa. This divergence suggests the involvement of these genes in responding to alfalfa leaf blight and stomach tissue pathological changes in mice, as well as in regulating disease resistance.

**Figure 4:**
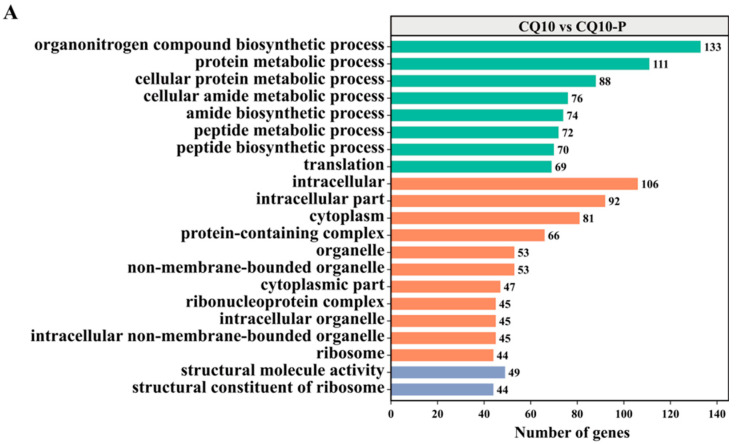

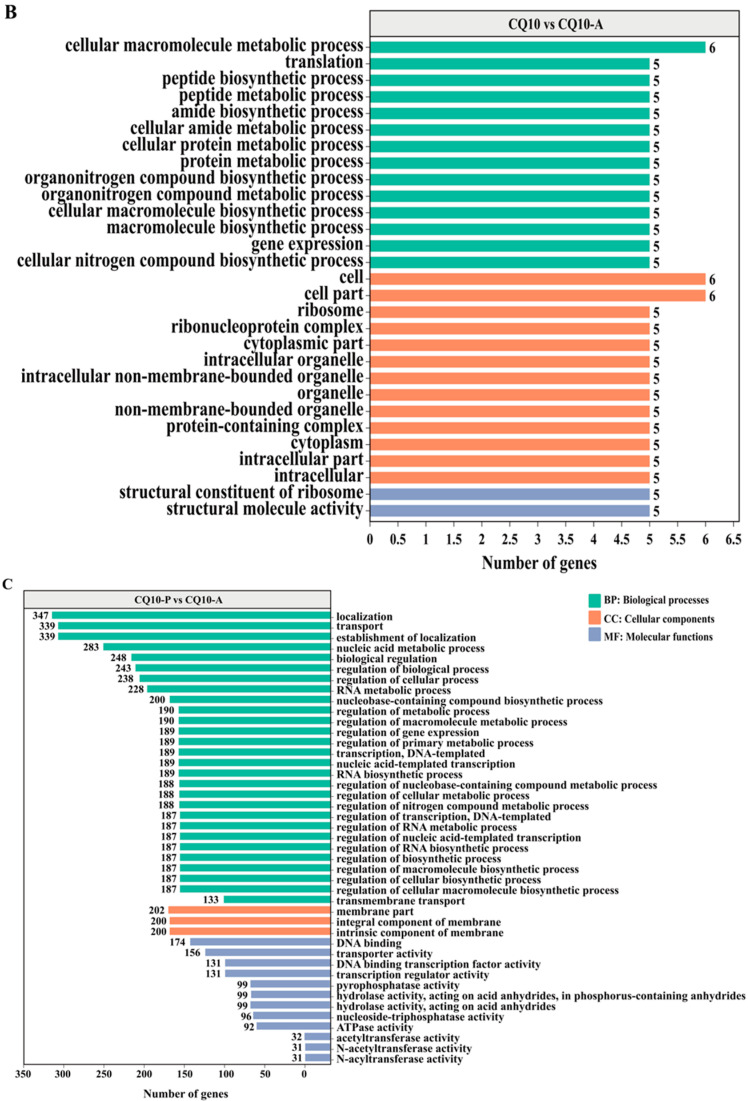
The top 30 GO annotated entries containing the upregulated DEGs. (**A**) The top 30 GO annotated entries containing the largest DEGs in the CQ10 group and CQ10-P group. (**B**) The 30 top GO annotated entries containing the largest DEGs in the CQ10 group and CQ10-A group. (**C**) The 30 top GO annotated entries containing the largest DEGs in the CQ10-P group and CQ10-A group.

To predict the interaction mechanism in the strain CQ10 infection of alfalfa and mice, GO was used to study the functional annotation of upregulated DEGs in the three GO branches, of which the top 30 GO annotated entries are shown in Figure 4. On the whole, the annotation of DEGs in the three GO branches of cellular components in the three groups was roughly the same, and was mainly involved in the cell, cell part, and intracellular part. In the biological process category of the CQ10/CQ10-P groups, DEGs were mainly involved in the cellular nitrogen compound biosynthetic process, the organonitrogen compound biosynthetic process, and the cellular macromolecule metabolic process. In the molecular function of the CQ10/CQ10-P groups, DEGs were mainly involved in binding and oxidoreductase activity (Figure 4A). In the biological process category of the CQ10/CQ10-A groups, DEGs were mainly involved in the cellular macromolecule metabolic process and translation. In the molecular function of the CQ10/CQ10-A groups, DEGs were mainly involved in the structural constituent of ribosome activity and structural molecule activity (Figure 4B). In the biological process category of the CQ10-P/CQ10-A groups, DEGs were mainly involved in the oxidation reduction process and the nucleobase-containing compound biosynthetic process. In the molecular function of the CQ10-P/CQ10-A groups, DEGs were mainly involved in ion binding and oxidoreductase activity (Figure 4C). These results implied that molecule binding, including the binding of small-molecule and macromolecular substances, played an important role in the interaction mechanism.

**Figure 5:**
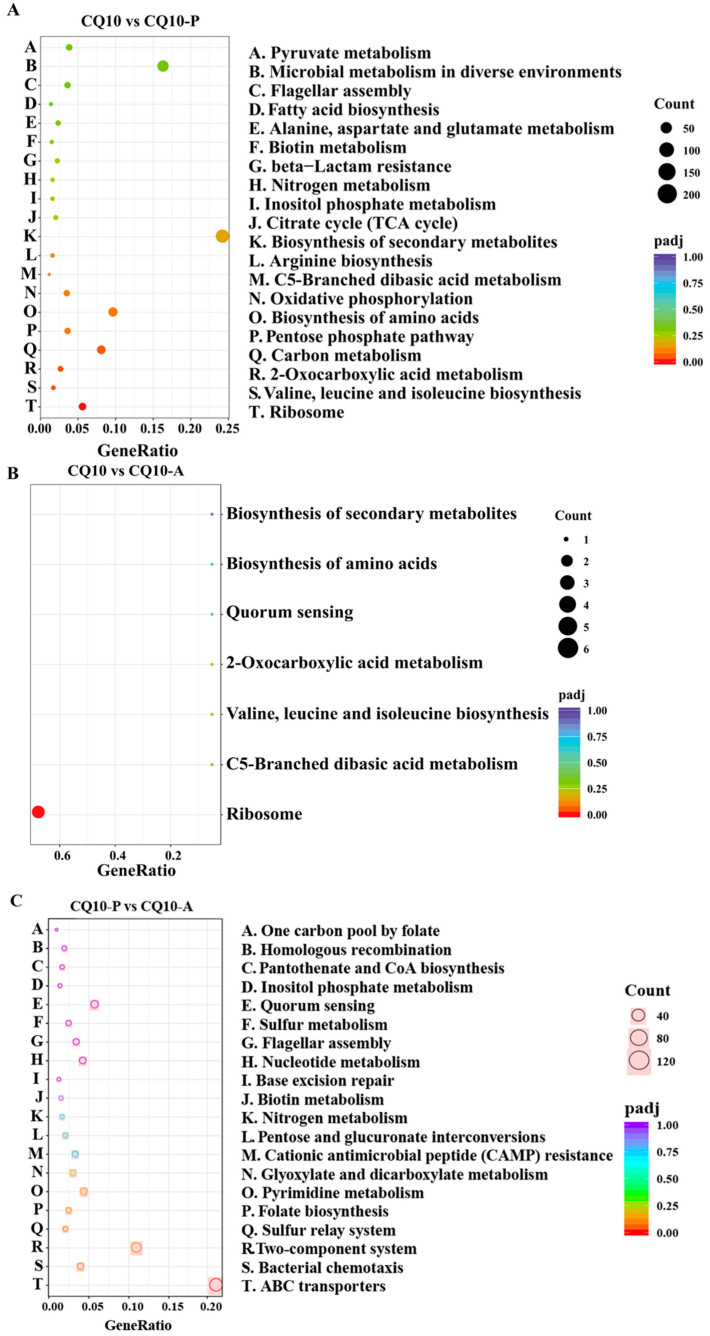
The top 30 KEGG pathways containing the upregulated DEGs. (**A**) The KEGG pathways containing the largest DEGs in the CQ10 group and CQ10-P group. (**B**) The KEGG pathways containing the largest DEGs in the CQ10 group and CQ10-A group. (**C**) The KEGG pathways containing the largest DEGs in the CQ10-P group and CQ10-A group. Circles indicate the count of DEGs.

KEGG pathway enrichment analysis was performed on all DEGs to investigate the effects of strain CQ10 expression on the enriched pathways of DEGs in alfalfa and mouse hosts. The top 20 pathways were identified (Figure 5A–C). Herein, metabolism and genetic-information-processing-related pathways were mainly enriched, including the “ribosome”, “biosynthesis of secondary metabolism”, and “ABC transporters” pathways. In particular, the “biosynthesis of secondary metabolism” and “microbial metabolism in diverse environments” pathway was only enriched in the CQ10/CQ10-P groups. However, “ribosome” was significantly enriched in the CQ10/CQ10-A groups and the CQ10-P/CQ10-A groups.

### 3.8. Toxicity Factor Analysis of DEGs

**Figure 6:**
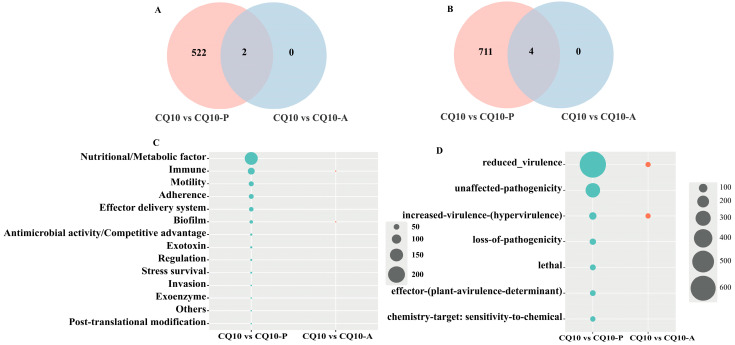
Toxicity factor analysis and pathogen–host interaction analysis of DEGs. (**A**) Venn analysis of virulence factors encoded by DEGs. (**B**) Venn analysis of pathogenic genes matched by DEGs in PHI-base. (**C**) The 14 pathways of virulence factors encoded by DEGs. (**D**) The 7 pathways of pathogenic genes matched by DEGs in PHI-base. Circles indicate the number of DEGs.

Virulence factors were identified by sequence alignment to the Virulence Factor of Bacterial Pathogens Database (VFDB: http://www.mgc.ac.cn/cgi-bin/VFs/v5/main.cgi, accessed on 10 November 2022) and binned into functional groups defined by VFDB [24]. The results revealed that a total of 714 virulence genes were identified in the DEGs of the CQ10 strain in alfalfa, accounting for 30.54%. However, only two virulence genes were identified in the DEGs of the CQ10 strain in mice, accounting for 4.65%. Specifically, the virulence factors of the CQ10 strain in alfalfa primarily involved the nutritional/metabolic factor (243, 34.03%), immune (117, 16.39%), motility (80, 11.20%), adherence (75, 10.50%), effector delivery system (67, 9.38%), and biofilm (48, 6.72%) factors. The virulence factors of the CQ10 strain in mice primarily encompassed immune and biofilm (Figure 6C) factors. Further comparison of the virulence factors encoded by DEGs across samples revealed that biofilm-associated protein (*Bap*) and *NAP* were consistently expressed by the CQ10 strain in both alfalfa and mouse hosts. *Bap* contributes to biofilm formation and adhesion, while *NAP* is linked to immune modulation [25] (Figure 6A). These findings indicate that biofilm formation and *NAP* play a significant role in the process of CQ10 strain infection alfalfa and mice.

### 3.9. Analysis of DEGs in Pathogenic Bacteria–Host Interactions

DEGs were queried against Pathogen Host Interactions (PHI-base: http://www.phi-base.org/, accessed on 11 November 2022) to identify pathogenesis-related genes and functions [26]. The results of functional annotation showed that 1055 pathogenic genes were identified in the DEGs of the CQ10 strain in alfalfa, accounting for 45.12%. Among them, 60.28% of genes were grouped in the category “reduced virulence”, 26.64% of genes in “molecular function”, and 3.32% of genes in “loss of pathogenicity”. In addition, only four pathogenesis-related genes were identified in the DEGs of the CQ10 strain in mice, of which three genes were classified in the category “reduced virulence”, and one gene was classified in “increased pathogenicity” (Figure 6D). Comparative analysis of pathogenic genes in the CQ10 strain when intercropped with alfalfa and mouse hosts revealed four co-expression genes: *PA2146*, *LeuB*, *EadM*, and *Dps* (atu2477) (Figure 6B). PA1246 was categorized as the category of increased pathogenicity, while the other three were categorized as the category of reduced virulence. These findings indicate that these genes have a crucial role in the process of the CQ10 strain infecting alfalfa and mice.

### 3.10. RNA-Seq Data Validation

RTq-PCR was performed to validate the RNA-seq results including eight genes that had different expression patterns. Among them, the expression of *rpsS*, *K6R05_RS11135*, *tssB*, and *rplP* were significantly upregulated, and the expression of *K6R05_RS18735*, *K6R05_RS17085*, *ssrA*, and *K6R05_RS19660* were significantly downregulated, but all of them were highly corrected by the RNA-seq results (Figure S3). These results further proved that the transcriptome data were reliable.

### 3.11. The Qualitative Proteome Analysis

To ensure the quality of proteomic analysis, the generated MS data were assessed and mainly examined for the distribution of protein sequence coverage (Figure S6). The high distribution of protein coverage showed that the identification results were accurate and highly reliable. The genome of 1246434-*Pantoea agglomerans*-*Medicago sativa* fasta was used as the proteins reference genome of the CQ10-P group. The genome of 1246434-*Pantoea agglomerans*-mouse fasta was used as the protein reference genome of the CQ10-A group. Proteomic analysis revealed that a total of 398,314 spectra, 51,954 peptides, and 9686 proteins were obtained from two samples (Table S3). Based on the transcriptome analysis, it was found that the gene expression level of CQ10 in mice was lower than that in alfalfa, and only six proteins were commonly identified across the two groups (Figure S6). Also, only the expression of the *Lpp* gene was consistent with the transcriptome. However, the other five DEPs can also be matched in the transcriptome (Table S4). Consequently, a comparison of DEPs and function were not feasible.

We analyzed the signaling pathways using GO and KEGG analysis. On the whole, the annotation of proteins in the three GO branches in two groups were roughly the same, differing only in the number of proteins (Figure S7). In the biological process category, proteins were mainly involved in the oxidation reduction process, the metabolic process, and translation. In molecular function, proteins were mainly involved in ATP binding, oxidoreductase activity, and the structural constituent of the ribosome. In addition, proteins were mainly involved in the membrane, intracellular, and intracellular in molecular function categories (Figure S7A,B). KEGG enrichment analysis showed that these proteins were involved in multiple metabolism pathways, including information on metabolism, genes, cellular processes, the environmental information process, and the organismal process. In the metabolism category of the CQ10-P and CQ10-A groups, proteins were mainly involved in global and overview maps, carbohydrate metabolism, and amino acid metabolism. In the genetic information processing of the CQ10-P group, proteins were mainly involved in transcription, folding, sorting, and degradation. In the CQ10-A group, proteins were mainly involved in signal transduction, signaling molecules, and interaction. In the cellular processes of the CQ10-A group, proteins were mainly involved in transport and catabolism and cellular community eukaryotes. In the environmental information processing of the CQ10-A group, proteins were mainly involved in translation, folding, sorting, and degradation. In the organismal systems of the CQ10-A group, proteins were mainly involved in the immune system, the endocrine system, and the digestive system (Figure S7C,D).

### 3.12. Genes and Proteins Related to CQ10 on Alfalfa and Mouse Cross-Kingdom Infection

To examine the congruence between the transcriptome and proteome, we conducted a comparative analysis between the protein and mRNA data. In total, 893 proteins matched to the transcripts. In the CQ10-P group, 879 proteins were matched, while in the CQ10-A group, 4 proteins were matched (Figure S8). We also performed a coherence annotation of the GO and KEGG enrichment analysis of all DEGs and DEPs. In the molecular function of the CQ10-P/CQ10-A groups, DEGs/DEPs were mainly involved in the structural constituent of the ribosome. In the biological process category of the CQ10-P/CQ10-A groups, DEGs/DEPs were mainly involved in translation. In the cellular component category of the CQ10-P/CQ10-A groups, DEGs/DEPs were mainly involved in ribosome, intracellular, and cytoplasm. The joint two omics KEGG pathway enrichment results revealed that DEGs/DEPs were mainly involved in the biosynthesis of secondary metabolism in the CQ10-P group. Additionally, DEGs/DEPs were mainly involved in the metabolism of other amino acids, the biosynthesis of other secondary metabolism, cell motility, and the immune system. These results implied that “ribosome”, secondary metabolites of bacteria, and bacterial motility played an important role in alfalfa and mouse cross-kingdom infection of CQ10.

## 4. Discussion

### 4.1. Strain CQ10 Is a Plant and Animal Cross-Kingdom Pathogenic Bacteria

Among the 24 species of *Pantoea* sp. that have been published, 7 species of bacteria are pathogenic to animal hosts, and some pathogenic bacteria have the ability to infect both animal and plant hosts to cause diseases, such as *Pantoea agglomerans* [27,28], *Pantoea ananatis* [29,30], *Pantoea dispersa* [31,32], and *Pantoea eucrina* [33]. In this study, the feed intake and cumulative growth rate of the body weight of mice in the CQ10 group were reduced compared with the CK group during the 72 h period of intragastric administration. Additionally, the result indicated a statistically significant difference at 24 h, consistent with numerous studies in animal hosts infected by various pathogenic bacteria [34,35,36]. Further, the spleen, lung, and stomach coefficients of the CQ10 group significantly increased, and counts of white blood cells, red blood cells, neutrophils, monocytes, and lymphocytes showed an increasing trend compared with the CK group at 48 h. These results suggest that the infection of the CQ10 strain leads to the enlargement of the spleen, lungs, and stomach and activated innate immune responses and defense signals in mice [37]. These enlarged tissues indicate that the immune system is reacting to infection or inflammation in mice [38]. Importantly, neutrophils, lymphocytes, and monocytes are considered primary effector cells of antimicrobial defense [39]. These neutrophils, lymphocytes, and monocytes are recruited to the site of infection, where they participate in clearing the pathogens, such as *Listeria monocytogenes*, *Legionella pneumophila*, and *Klebsiella pneumoniae* [40,41].

Histopathological sections can intuitively reflect the degree of organ damage [42]. This study found that stomach, liver, spleen, and kidney tissues appeared to have severe pathological symptoms after oral gavage of CQ10 suspension to mice; whereas, the pathological symptoms in the heart and lungs were relatively mild. Among them, a part of the gastric mucosal cells was mainly damaged by the CQ10 strain in the stomach. It was exhibited that the CQ10 strain may adhere to and localize to the surface epithelial cells of the gastric mucosa after bacteria enter the stomach, potentially leading to further inflammatory reactions. The ongoing inflammatory response leads to the damage of the gastric mucosa. Bacterial adherence to epithelial cell surfaces is believed to be an important first step in the initiation of infection, followed by the activation of a range of chemokines and cytokines, leading to the induction of inflammatory lesions in the gastric mucosa [43]. The liver is a frontline innate immunologic and essential metabolic organ that plays a major role in the detection, capture, and clearance of pathogens. Upon invasion by a pathogen, the liver transitions from a state of low immune response to one of intense inflammatory response and effective adaptive immunity, thereby producing an oxidative stress injury [44]. In this study, the infection of the CQ10 strain caused pathological changes in the liver tissues of mice. This may be due to the inflammatory response triggered by bacterial cells or their metabolites entering the liver tissues. Moreover, the spleen is a crucial infection site in which animal hosts respond to bacterial infection. For example, *Listeria monocytogenes* and *Salmonella* were able to colonize the spleen tissue while infecting mice [45]. Similarly, spleen tissues were pathologically damaged to different degrees in the CQ10 group. This indicates that the spleen might also be a location of colonization and infection by the CQ10 strain. The kidney is a component of the urinary system in animal hosts. Pathogenic bacteria, such as *Staphylococcus aureus*, can enter the urinary system via the gastrointestinal tract and cause infections. Notably, *S. aureus* has been observed to colonize the kidneys of mice, leading to widespread discoloration [46]. Furthermore, CQ10 strains primarily induce pathological changes in the glomerular and tubular epithelium of the mice’s kidneys. Cells matching the CQ10 strains were also isolated following the grinding of the kidney, further indicating that CQ10 can enter the kidneys of mice and cause harm.

Taken together, these results demonstrate that the CQ10 strain has the ability to cause the disease in mice. Significantly, CQ10 strains were isolated from the feces of mice at four different time points. This suggests that CQ10 enters animals through livestock feeding and is subsequently excreted into the soil, potentially initiating further infections. Drawing from the research outcomes of this laboratory [9], CQ10 is a plant and animal cross-kingdom pathogenic bacterium. The results of this study change our understanding of the evolution of this bacterium. Previous studies primarily focused on the pathogenicity of pathogenic bacteria towards a single host; whereas, this study aims to uncover the cross-kingdom pathogenicity of bacteria towards plants and animals at the single strain level. Since the origin of life on Earth, the diversification and success of microbes have been intimately intertwined with their physical and chemical environments [47]. On the one hand, with the current substantial changes in climate and trade, microorganisms also have more habitats and are well adapted, and their genomes are constantly transforming in response to environmental pressures [47]. On the other hand, the pathogenesis of plants and animals is in some ways identical [48]. The emergence of transboundary pathogenic microorganisms is, therefore, also the result of a continuous microbial succession. In addition, CQ10 strains were isolated from the feces of all inoculated mice. This is a strong indication that CQ10 will be circulated throughout the grassland agroecosystem by means of livestock feeding, entering the stomach and intestines of livestock, and then, entering the soil through fecal excretion. Whether it also poses a threat to other ecosystems will be subject to further study.

### 4.2. Genes Associated with the Plant and Animal Cross-Kingdom of Pathogenic Bacteria

Pathogenic bacteria are armed with diverse strategies to help their invasion and infection from the natural environment to the host. With the advance in molecular biology technologies, we took advantage of transcriptomic sequencing to analyze gene expression during bacterial infection, which has emerged as a new fundamental layer of control of gene expression [15]. In the study, we determined that CQ10 can cause the cross-kingdom infection of alfalfa and mice from the perspective of transcriptomics. A total of 20 DEGs were identified in alfalfa and mice inoculated with the CQ10 strain (Figure 3A), primarily enriched in GO pathways, such as “location”, “transport”, “establishment of location”, “membrane part”, and “DNA banding” (Figure S5). Among them, upregulated DEGs were primarily concentrated in pathways, such as “location”, “transport”, and “ion binding” (Figure 4). The KEGG pathway enriched by DEGs is “ribosome”, and all DEGs in this pathway are upregulated genes (Figure 4). It has been reported that pathogenic bacteria can modulate gene expression by the changes in the composition of ribosomes, indirectly regulating gene expression at the post-transcriptional level. For instance, *Francisella tularensis* can regulate the components of the Type VI secretion systems (T6SSs) [49]. Thus, we hypothesize that ribosomal pathways encoded by upregulated *rplP* and *rpsS* may serve a role in virulence expression and regulation. All of the aforementioned evidence points toward the conclusion that the CQ10 strain infection process is regulated by a complex metabolic network, mediated by signaling pathways, including “location”, “ion binding”, “membrane component”, and “ribosome”.

Intriguingly, we also found that DEGs were enriched in several toxicity factor pathways, including nutritional/metabolic factors, immune, motility, adherence, effector delivery system, and biofilm (Figure 5C). This is similar to the virulence factors relied upon by other cross-kingdom pathogenic bacteria [50]. This further shows that the CQ10 strain relies on diverse virulence factors during cross-kingdom infection and overcomes the challenges faced in adhesion, colonization, survival, and infection by coordinating with each other. Therefore, the efficient and orderly regulation of the expression of these virulence factors constitutes the key virulence strategy in cross-kingdom pathogenic CQ10. In addition, we found that “biofilm-associated protein (*Bap*)” and “neutrophil activation protein (*NAP*)” are enriched in alfalfa and mouse hosts (Figure 5A), suggesting that *Bap* and *NAP* play pathogenic roles for cross-kingdom pathogenic bacteria in the pathogenic process. Several in vitro experiments have illustrated that the *Bap* system was described to trigger cellular adhesion and positively impact biofilm formation [51], and the knockout of the gene encoding *Bap* was shown to miss the ability of the pathogenic bacteria to form biofilms; the *Bap*-complemented strain promoted biofilm production. Such experimental infection studies indicated that all the *Streptococcus* aureus strains harboring the *Bap* gene are strong biofilm producers, and the presence of *Bap* significantly increased the ability of *S. aureus* to colonize and persist in the mammary gland [52]. Intriguingly, a number of surface proteins exhibiting sequence similarities with *Bap* have recently been shown to be involved in biofilm formation in different bacterial species, such as *Burkholderia cepacian* [53], *Pseudomonas fluorescens* [54], and *Escherichia coli* [55]. Additionally, *Bap* can induce the formation of biofilms in the absence of exopolysaccharide (EPS) [56]. *Bap* may also participate in receptor recognition and enhance the colonization ability and the development of the infections of the strain in host cells [56]. Overall, these properties possessed by *Bap* are exactly those necessary for pathogenic bacteria to successfully infect a single host and expand their host range. Previous studies have demonstrated that *NAP* could induce and promote the adhesion between neutrophils and endothelial cells. For example, the *NAP* protein encoded by the pathogenic bacterium *Helicobacter pylori* primarily induces and enhances the adhesion between neutrophils and endothelial cells and the resulting massive infiltration of neutrophils, which intensifies the *H. pylori* infection of the gastric mucosa [57]. In addition, *NAP* can also activate monocytes and neutrophils and induce proinflammatory cytokines (IL-6, IL-12, TNF-α, IL-23) to promote inflammation [25]. It is evident that the *NAP* protein encoded by the CQ10 strain possibly plays a role in the infection process of animal hosts, since the same pathology was observed in the present study (Figure 5A). So, we propose that *NAP* could exercise this function, but further research is needed.

Moreover, DEGs were further validated in the PHI-base database. The PHI-base catalogues more than 2800 genes from fungi, bacteria, and protist pathogens, with experimentally verified pathogenicity, virulence, and effector genes [58]. In this study, we discovered that four pathogenic bacteria–host interactions genes were commonly enriched in both alfalfa and mice (Figure 5B). Among them, *LeuB* was encoded by 3-isopropylmalate dehydrogenase, which is a crucial enzyme for the biosynthesis of leucine. Ren et al. showed that the leucine biosynthesis pathways were found to be absent after *leuB* deletion in *Acidovorax citrulli*, resulting in reduced cell growth and motility, as well as a significant reduction in infection in the melons host (*Cucumis melo*) [59]. According to the KEGG enrichment result, the biosynthesis of the leucine pathway was enriched in the upregulated DEGs in the CQ10 strain. This implies that the biosynthesis of leucine plays a pivotal role in the growth and bacterial virulence of the CQ10 strain in animal and plant hosts. Next, *Dps* (atu2477) induces ferritins in *Agrobacterium tumefaciens*, a ferritin protein that maintains iron homeostasis; mutations in this gene cause the disruption of iron homeostasis and metabolism, and this improvement contributes to the decreased resistance of the mutant strain to oxidative stress [60]. Also of note is that the iron binding pathway was enriched in the upregulated DEGs in GO. This suggests that iron acquisition strategies may be another important aspect of the CQ10 strain in the growth and virulence of the CQ10 strain [61].

Hence, we speculate that *Bap* or homologous *Bap*-protein-mediated biofilm formation and iron acquisition are crucial factors in the ability of CQ10 to coinfect and cause disease across both the plant and animal kingdoms. Moreover, the morphology of CQ10 shows that it has a high capacity for biofilm formation, which plays an important role in its pathogenicity to plants and animals [62]. Of course, this does not suggest that all bacteria containing *Bap* protein or ferritin possess the ability to cause disease across the kingdom, but further research is needed.

### 4.3. Proteins Associated with the Plant and Animal Cross-Kingdom of Pathogenic Bacteria

Using proteomics technology, we have investigated differential expression between the two samples. As a result, a total of 9686 proteins were identified, of which 6 were significantly differentially expressed (FC ≤ 1%). Also, only the expression of the *lpp* gene was consistent with the transcriptome level; the other five DEPs can only be matched in the transcriptome (Table S4). The relatively lower number of differentially expressed genes might be attributed to the inconsistent expression of genes at the transcription and translation levels. Then, the virulence factors of cross-kingdom pathogenic bacteria are not consistent between plants and animals [63]. Bacterial *Lpp* is a class of membrane-anchored proteins that are found on the outer membrane of bacteria within the Enterobacteriaceae family, playing a role in the immune response to bacterial infection. It serves as a crucial virulence factor, playing a pivotal role in both the host and pathogen properties, engaging in bacterial adhesion, colonization, escaping the immune defense system of the host, and immune defense [64]. *Mycobacterium tuberculosis* lipoproteins *LprA* help bacteria escape from the host defenses through inducing the cytokine responses [65]. Recently, it was shown that the loss of *Lpp* of *Aeromonas veronii* JC529 decreased cell motility, the ability to form biofilm, and adhesive ability, as well as the virulence of the *Lpp* mutant strain being attenuated in the infection of mice [66]. This is consistent with transcriptomic analyses, where *Bap* also significantly increased the ability to form biofilm. This evidence fully indicates that biofilm is an important virulence factor of the CQ10 strain to coinfect and cause disease across both plant and animal kingdoms, and *Bap* and *Lpp* might be the key pathogenic gene of CQ10, but further research is needed. Compared with the article published by Rahme Laurence G in *Science* [8], this study increases the resources surrounding cross-kingdom pathogenic genes.

## 5. Conclusions

In this experiment, we established the alfalfa root infection model and mouse gavage model and clarified that CQ10 is a common plant and animal cross-kingdom pathogenic bacterium. CQ10 mainly causes bacterial leaf blight in alfalfa. Mice exhibited diminished vitality, tendencies to huddle, lack of appetite, and severe pathological changes in the stomach, liver, kidney, and spleen tissues. Moreover, we isolated CQ10 strains from mice feces at different inoculation times. Through dual RNA-seq and proteomic analysis, both DEGs and DEPs were enriched in the virulence of the biofilm. Among them, *Bap* and *lpp* are key cross-kingdom genes. These research findings can serve as a new reference direction for global pathogen management. Additionally, the gene resources obtained from this study lay a solid foundation for the subsequent functional verification of virulence factors in cross-kingdom pathogenic bacteria.

## Figures and Tables

**Table 1:** The sample used for transcriptome sequencing.

Samples	Group	Type
CQ10-1	CQ10	10^9^ cfu/mL bacteria strain
CQ10-2		
CQ10-3		
CQ10-P-1	CQ10-P	Alfalfa leaves infested with 10^9^ cfu/mL CQ10 strain
CQ10-P-2		
CQ10-P-3		
CQ10-A-1	CQ10-A	Stomach tissue of mice infested with 10^9^ cfu/mL CQ10 strain
CQ10-A-2		
CQ10-A-3		

**Table 2:** The sample used for proteome sequencing.

Samples	Group	Type
CQ10-P-1	CQ10-Plant	Alfalfa leaves infested with 10^9^ cfu/mL CQ10 strain
CQ10-P-2		
CQ10-P-3		
CQ10-A-1	CQ10-Animal	Stomach tissue of mice infested with 10^9^ cfu/mL CQ10 strain
CQ10-A-2		
CQ10-A-3		

## Data Availability

Data will be made available on request.

## Supplementary Materials

The following supporting information can be downloaded at: https://www.mdpi.com/article/10.3390/microorganisms12112197/s1, Figure S1: A Platelet (PLT) counts. B Hb hemoglobin (HGB) counts. C The red blood cell distribution width coefficient of variation (RDW). D Hematocrit (HCT). E Mean cell volume (MCV). F Mean corpuscular hemoglobin (MCH) concentration; Figure S2: Clustering analysis by total DEGs. The horizontal coordinates refer to the sample group, and the vertical coordinates refer to the expression of DEGs. Red for upregulation and blue for downregulation; Figure S3: RTq-PCR verification of DEGs. A RTq-PCR verification of upregulated genes. B RTq-PCR verification of downregulated genes; Figure S4: Evaluation results of proteomic quality control. A The distribution of protein coverage of the CQ10-P group. B The distribution of protein coverage of the CQ10-A group; Figure S5: The GO annotated entries containing the largest DEGs. A, The top 20 GO annotated entries containing the largest DEGs in the CQ10 group and CQ10-P group. B, The top 20 GO annotated entries containing the largest DEGs in the CQ10-P group and CQ10-A group. C, The GO annotated entries containing the largest DEGs in the CQ10 group and CQ10-A group; Figure S6: Venn analysis of number of DEPs; Figure S7: The GO annotated entries and KEGG pathways containing the largest DEPs. A, The top 30 GO annotated entries containing the largest DEPs in the CQ10-P group. B, The 30 top GO annotated entries containing the largest DEPs in the CQ10-A group. C, The KEGG pathways containing the largest DEPs in the CQ10-P group. D, The KEGG pathways containing the largest DEPs in the CQ10-A group; Figure S8: Joint Venn diagram analysis for total DEGs/DEPs; Table S1: Sequence of RTq-PCR primers; Table S2: The results of the comparison of reads to the reference genome; Table S3: Protein Identification Overview; Table S4: Co-expressed protein in alfalfa and mice.

## References

[1] Banerjee S., van der Heijden M.G.A. (2023). Soil microbiomes and one health. Nat. Rev. Microbiol..

[2] Berg G., Rybakova D., Fischer D., Cernava T., Vergès M.C., Charles T., Chen X., Cocolin L., Eversole K., Corral G.H. (2020). Microbiome definition re-visited: Old concepts and new challenges. Microbiome.

[3] Hou F.J., Xu L. (2010). Meaning of the animal production layer in grassland agroecosystems. Pratacultural Sci..

[4] Fones H.N., Bebber D.P., Chaloner T.M., Kay W.T., Steinberg G., Gurr S.J. (2020). Threats to global food security from emerging fungal and oomycete crop pathogens. Nat. Food.

[5] Abbas A., Mubeen M., Sohail M.A., Solanki M.K., Hussain B., Nosheen S., Kashyap B.K., Zhou L., Fang X. (2022). Root rot a silent alfalfa killer in China: Distribution, fungal, and oomycete pathogens, impact of climatic factors and its management. Front. Microbiol..

[6] Tammam S.N., El Safy S., Ramadan S., Arjune S., Krakor E., Mathur S. (2021). Repurpose but also (nano)-reformulate! The potential role of nanomedicine in the battle against SARS-CoV2. J. Control Release.

[7] Nan Z.B., Li C.J., Bai Y.S. (2003). Diseases of Grass Crops in China and Their Control Countermeasures.

[8] Rahme L.G., Stevens E.J., Wolfort S.F., Shao J., Tompkins R.G., Ausubel F.M. (1995). Common virulence factors for bacterial pathogenicity in plants and animals. Science.

[9] Yao B., Huang R., Zhang Z.Z., Shi S.L. (2022). Seed-Borne *Erwinia persicina* Affects the Growth and Physiology of Alfalfa (*Medicago sativa* L.). Front. Microbiol..

[10] Zhang Z.F., Nan Z.B. (2014). *Erwinia persicina*, a possible new necrosis and wilt threat to forage or grain legumes production. Eur. J. Plant Pathol..

[11] Zhang Z.F. (2013). Seed-Borne Bacteria of Lucerne (*Medicago sativa*) and Their Pathogenicity. Ph.D. Thesis.

[12] Mohamaden W.I., Zhang Z.F., Hegab I.M., Shi S.L. (2019). Experimental infection in mouse with *Erwinia persicina*. Microb. Pathog..

[13] Elvira-Recuenco M., van Vuurde J.W. (2000). Natural incidence of endophytic bacteria in pea cultivars under field conditions. Can. J. Microbiol..

[14] Cruz A.T., Cazacu A.C., Allen C.H. (2007). *Pantoea agglomerans*, a plant pathogen causing human disease. J. Clin. Virol. Plus.

[15] Finet O., Yague-Sanz C., Krüger L.K., Tran P., Migeot V., Louski M., Nevers A., Rougemaille M., Sun J., Ernst F.G.M. (2022). Transcription-wide mapping of dihydrouridine reveals that mRNA dihydrouridylation is required for meiotic chromosome segregation. Mol. Cell..

[16] Lum K.K., Cristea I.M. (2016). Proteomic approaches to uncovering virus-host protein interactions during the progression of viral infection. Expert. Rev. Proteom..

[17] Wu M.X., Zou Y., Yu Y.H., Chen B.X., Zheng Q.W., Ye Z.W., Guo L.Q., Lin J.F. (2021). Comparative transcriptome and proteome provide new insights into the regulatory mechanisms of the postharvest deterioration of *Pleurotus tuoliensis* fruitbodies during storage. Food Res. Int..

[18] Qu S., Li M., Wang G., Yu W., Zhu S. (2021). Transcriptomic, proteomic and LC-MS analyses reveal anthocyanin biosynthesis during litchi pericarp browning. Sci. Hortic..

[19] Yao B., Zhang Z.F., Shi S.L., Zhang Y.J., Jia Q.Y., Li X.Y., Zhang H., Deji Z.M. (2021). A Plant Seed Germination and Seedling Stress Experimental Device.

[20] Zhu S., Xie J., Yang J., Hou X., He L., Zhang Z. (2024). Seed-Borne Bacterial Diversity of Fescue (*Festuca ovina* L.) and Properties Study. Microorganisms.

[21] Kachuk C., Stephen K., Doucette A. (2015). Comparison of sodium dodecyl sulfate depletion techniques for proteome analysis by mass spectrometry. J. Chromatogr. A.

[22] Sun Z., Li L., Qu J., Li H., Chen H. (2019). Proteomic analysis of therapeutic effects of Qingyi pellet on rodent severe acute pancreatitis-associated lung injury. Biomed. Pharmacother..

[23] Livak K.J., Schmittgen T.D. (2001). Analysis of relative gene expression data using real-time quantitative PCR and the 2 (-Delta Delta C(T)) method. Methods.

[24] Chen L., Zheng D., Liu B., Yang J., Jin Q. (2016). VFDB 2016: Hierarchical and refined dataset for big data analysis-10 years on. Nucleic Acids Res..

[25] Amedei A., Cappon A., Codolo G., Cabrelle A., Polenghi A., Benagiano M., Tasca E., Azzurri A., D’Elios M.M., Del Prete G. (2006). The neutrophil-activating protein of *Helicobacter pylori* promotes Th1 immune responses. J. Clin. Investig..

[26] Consortium R. (2021). RNA central 2021: Secondary structure integration, improved sequence search and new member databases. Nucleic Acids Res..

[27] Liberto M.C., Matera G., Puccio R., Lo Russo T., Colosimo E., Focà E. (2009). Six cases of sepsis caused by *Pantoea agglomerans* in a teaching hospital. New Microbiol..

[28] Choi O., Kim H., Lee Y., Kim J., Moon j., Hwang I. (2012). First report of sheath rot of rice caused by *Pantoea ananatis* in Korea. Plant Pathol. J..

[29] De Armas S., Galván G.A., Lapaz M.I., González-Barrios P., Vicente E., Pianzzola M.J., Siri M.I. (2022). Phylogeny and Identification of Pantoea Species Associated with Bulb Rot and Bacterial Leaf Blight of Onion Crops in Uruguay. Plant Dis..

[30] Guajardo J., Vasconez I.N., Dorta F., Cuadros F., Yañez C., Valenzuela M. (2023). First Report of *Pantoea ananatis* and *Pantoea eucalypti* causing onion leaf blight and bulb decay in Central Chile. Plant Dis..

[31] Ilyas N., Yang Y., Liu W., Li X., Pu W., Singh R.P.P., Li Y. (2021). First report of bacterial rot caused by *Pantoea endophytica* on tobacco in Liuyang, China. Plant Dis..

[32] Wadhwa N., Berg H.C. (2022). Bacterial motility: Machinery and mechanisms. Nat. Rev. Microbiol..

[33] Tans-Kersten J., Huang H., Allen C. (2001). *Ralstonia solanacearum* needs motility for invasive virulence on tomato. J. Bacterioli.

[34] Dietrich C., Heuner K., Brand B., Hacker J., Steinert M. (2001). Flagellum of *Legionella pneumophila* positively affects the early phase of infection of eukaryotic host cells. Infect. Immun..

[35] Praveena P.E., Periasamy S., Kumar A.A., Singh N. (2010). Cytokine profiles, apoptosis and pathology of experimental *Pasteurella multocida* serotype A1 infection in mouse. Res. Vet. Sci..

[36] Van Heeckeren A.M., Tscheikuna J., Walenga R.W., Konstan M.W., Davis P.B., Erokwu B., Haxhiu M.A., Ferkol T.W. (2000). Effect of *Pseudomonas* infection on weight loss, lung mechanics, and cytokines in mouse. Am. J. Respir. Crit. Care Med..

[37] Allemailem K.S., Alnuqaydan A.M., Almatroudi A., Almatroudi A., Alrumaihi F., Aljaghwani A., Khalilullah H., Younus H., Khan A., Khan M.A. (2021). Safety and therapeutic efficacy of thymoquinone-loaded liposomes against drug-sensitive and drug-resistant *Acinetobacter baumannii*. Pharmaceutics.

[38] Kim J.H., Lim I.R., Joo H.J., Park C.Y., Choi S.C., Jeong H.S., Hong S.J. (2019). Fimasartan reduces neointimal formation and inflammation after carotid arterial injury in apolipoprotein E knockout mouse. Mol. Med..

[39] Kaur G., STS C., Nimker C., Bansal A. (2015). rIL-22 as an adjuvant enhances the immunogenicity of rGroEL in mouse and its protective efficacy against *S. Typhi* and *S. Typhimurium*. Cell Mol. Immunol..

[40] Casson C.N., Doerner J.L., Copenhaver A.M., Ramirez J., Holmgren A.M., Boyer M.A., Siddarthan I.J., Rouhanifard S.H., Raj A., Shin S. (2017). Neutrophils and Ly6Chi monocytes collaborate in generating an optimal cytokine response that protects against pulmonary *Legionella pneumophila* infection. PLoS Pathog..

[41] Xiong H., Carter R.A., Leiner I.M., Tang Y.W., Chen L., Kreiswirth B.N., Pamer E.G. (2015). Distinct contributions of neutrophils and CCR2+ monocytes to pulmonary clearance of different *Klebsiella pneumoniae* strains. Infect. Immun..

[42] Luo H., Gu C., Liu C., Wang Y., Wang H., Li Y. (2018). Plasma metabolic profiling analysis of *Strychnos nux-vomica* Linn. and *Tripterygium wilfordii* Hook F-induced renal toxicity using metabolomics coupled with UPLC/Q-TOF-MS. Toxicol. Res..

[43] Suzuki H., Masaoka T., Miyazawa M., Suzuki M., Miura S., Ishii H. (2002). Gastric mucosal response to Helicobacter pylori. Keio J. Med..

[44] Kubes P., Jenne C. (2018). Immune responses in the liver. Annu. Rev. Immunol..

[45] Beristain-Covarrubias N., Perez-Toledo M., Flores-Langarica A., Zuidscherwoude M., Hitchcock J.R., Channell W.M., King L.D.W., Thomas M.R., Henderson I.R., Rayes J. (2019). Salmonella-induced thrombi in mouse develop asynchronously in the spleen and liver and are not effective bacterial traps. Blood.

[46] Abraham S.N., Miao Y. (2015). The nature of immune responses to urinary tract infections. Nat. Rev. Immunol..

[47] Jaffe A.L., Castelle C.J., Banfield J.F. (2023). Habitat Transition in the Evolution of Bacteria and Archaea. Annu. Rev. Microbiol..

[48] Ali R., Ma W., Lemtiri-Chlieh F., Tsaltas D., Leng Q., von Bodman S., Berkowitz G.A. (2007). Death don’t have no mercy and neither does calcium: Arabidopsis CYCLIC NUCLEOTIDE GATED CHANNEL2 and innate immunity. Plant Cell.

[49] Trautmann H.S., Ramsey K.M. (2002). A ribosomal protein homolog governs gene expression and virulence in a bacterial pathogen. J. Bacteriol..

[50] Pakbin B., Brück W.M., Rossen J.W.A. (2021). Virulence factors of enteric pathogenic *Escherichia coli*: A review. Int. J. Mol. Sci..

[51] Taglialegna A., Navarro S.L., Ventura S., Garnett J.A., Matthews S., Penades J.R., Lasa I., Valle J. (2016). Staphylococcal Bap proteins build amyloid scaffold biofilm matrices in response to environmental signals. PLoS Pathog..

[52] Hinsa S.M., Espinosa-Urgel M., Ramos J.L., O’Toole G.A. (2003). Transition from reversible to irreversible attachment during biofilm formation by *Pseudomonas fluorescens* WCS365 requires an ABC transporter and a large secreted protein. Mol. Microbiol..

[53] Cucarella C., Tormo M.Á., Úbeda C., Trotonda M.P., Monzón M., Peris C., Amorena B., Lasa I., Penadés J.R. (2004). Role of biofilm-associated protein bap in the pathogenesis of bovine *Staphylococcus aureus*. Infect. Immun..

[54] Huber B., Riedel K., Köthe M., Givskov M., Molin S., Eberl L. (2009). Genetic analysis of functions involved in the late stages of biofilm development in *Burkholderia cepacia* H111. Mol. Microbiol..

[55] Roux A., Beloin C., Ghigo J.M. (2005). Combined inactivation and expression strategy to study gene function under physiological conditions: Application to identification of new *Escherichia coli* adhesins. J. Bacteriol..

[56] Lasa I., Penadés J.R. (2006). Bap: A family of surface proteins involved in biofilm formation. Res. Microbiol..

[57] Satin B., del Giudice G., Bianca V.D., Dusi S., Laudanna C., Tonello F., Kelleher D., Rappuoli R., Montecucco C., Rossi F. (2000). The neutrophil-activating protein (HP-NAP) of *Helicobacter pylori* is a protective antigen and a major virulence factor. J. Exp. Med..

[58] Liu F., Ou X.C., Zhan R.L. (2018). Whole-genome sequence and genome annotation of *Xanthomonas citri* pv. mangiferaeindicae, causal agent of bacterial black spot on Mangifera indica. Arch. Microbiol..

[59] Ren Z.G., Jiang W.J., Ni X.Y., Lin M., Zhang W., Tian G.Z., Zhang L.Q. (2014). Multiplication of *Acidovorax citrulli* in planta during infection of melon seedlings requires the ability to synthesize leucine. Plant Pathol..

[60] Yang J., Pan X., Xu Y., Li Y., Xu N., Huang Z., Ye J., Gao D., Guo M. (2020). Agrobacterium tumefaciens ferritins play an important role in full virulence through regulating iron homeostasis and oxidative stress survival. Mol. Plant Pathol..

[61] Fusco W.G., Choudhary N.R., Council S.E., Collins E.J., Leduc I. (2013). Mutational analysis of hemoglobin binding and heme utilization by a bacterial hemoglobin receptor. J. Bacteriol..

[62] Hall-Stoodley L., Costerton J.W., Stoodley P. (2004). Bacterial biofilms: From the Natural environment to infectious diseases. Nat. Rev. Microbiol..

[63] Büttner D., Bonas U. (2003). Common infection strategies of plant and animal pathogenic bacteria. Curr. Opin. Plant Biol..

[64] Iwasaki A., Medzhitov R. (2010). Regulation of adaptive immunity by the innate immune system. Science.

[65] Pietras E.M., Miller L.S., Johnson C.T., O’Connell R.M., Dempsey P.W., Cheng G. (2011). A MyD88-dependent IFNγR-CCR2 signaling circuit is required for mobilization of monocytes and host defense against systemic bacterial challenge. Cell Res..

[66] Sheng T., Song G., Yue T., Zhang J., Wang W., Yang Z., Lu Q. (2021). Whole-genome sequencing and antimicrobial resistance analysis of multidrug-resistant *Aeromonas veronii* strain JC529 from a common carp. J. Glob. Antimicrob. Resist..

