# Supplementary material for: Cross-Kingdom Pathogenesis of Pantoea alfalfae CQ10: Insights from Transcriptome and Proteome Analyses
Source: Microorganisms. 2024 Oct 30;12(11):2197. doi: 10.3390/microorganisms12112197 (PMC11596184; doi:10.3390/microorganisms12112197)
Supplement: Supplementary file 1 [file microorganisms-12-02197-s001.zip › microorganisms-3230684-supplementary.pdf]

Table S1: Sequence of RTq-PCR primers.

Gene	Forward primer（5`-3`）	Reverse primer（5`-3`）
K6R05_RS11135	CGCTGGAAGATGTGCTGGTGAC	GCATAAGTTACGCCCAGGGTGTC
K6R05_RS11145	CGACGACCGCAAATTCCTTGAAATC	GGCGAGAAGTCATCCATGCTGTC
K6R05_RS18735	GCAGCAGGTGAGAAGAGTGATTGAC	TTCGTGGTGACTTTGAGCAGATCG
K6R05_RS17085	TAGTACAGGTCGTGGCTGCTCATC	GCAATCATCCCGCCCAGAATACC
K6R05_RS04870	CGACGGGATTGTGAAGCCTTAGG	CAGACACGCCACTAACAGACTAGC
K6R05_RS19660	GTGAAGCTGGCAGTGCAGGAG	TTAGAAGGACGGCGACTAATCTTGC
K6R05_RS02175	CGGATGTCAGCTTCGGTACTTTCG	CGGCTTCTCGGTGATCGGTTTG
K6R05_RS02160	CCTGCACTTGCTGAAGAAGGTAGAG	CACCCAGTTTGTGACCAACCATTTC
K6L24_RS05045	GCAAACGACGAAAACTACGCACTAG	AACAGACACGCCACTGACAAACTAG
K6L24_RS13355	TGGGCGTTAAGCAGGAAGAAGTTG	ATCAATAGCAGCCTGAACGGTAGTG
K6L24_RS22220	CCCAGTCTCGTGAAGTGTCTTTAGC	CGATACCGTCTTCCAGCAGCATC
K6L24_RS06145	GCACGGCGGGCTGAAGATATTC	CCCTGATAACCACTGAGCAGCATTC
K6L24_RS00700	TTTCAACGGACCTGGGTTTCTCTTC	GGCGATACTGGATACGGTAATCACG
K6L24_RS01060	GCTCAACGACGGCACAAAGATTTC	TGGCTCAACAAGACCTTCAACACC
K6L24_RS09815	AGTCACATTGCTAACGGCGATATGG	GATACTGGCGACAATACCGAAGACC
K6L24_RS16845	ACCGAGTGGACATCCGAAGAGTAG	TCGCCTTCACACTTGCCTTCAC
rnpB	CTTCGACAGCATGGATGACTTCTC	GCACCTTACCAATCAGCTCTTCAG

Table S2: The results of the comparison of reads to the reference genome.

Sample	Total reads	Total mapped	Multiple mapped	Uniquely mapped
CQ10-1	20812614	19989906 (96.05%)	453142 (2.18%)	19536764 (93.87%)
CQ10-2	14684750	13558214 (92.33%)	662413 (4.51%)	12895801 (87.82%)
CQ10-3	18924412	18277193 (96.58%)	662094 (3.50%)	17615099 (93.08%)
CQ10-P-1	56201440	1433949 (2.55%)	35418 (0.06%)	1398531 (2.49%)
CQ10-P-2	91833014	3054952 (3.33%)	87234 (0.09%)	2967718 (3.23%)
CQ10-P-3	81061672	2549543 (3.15%)	58529 (0.07%)	2491014 (3.07%)
CQ10-A-1	89465358	94353 (0.11%)	94324 (0.11%)	29 (0%)
CQ10-A-2	93772256	109541 (0.12%)	109498 (0.12%)	43 (0%)
CQ10-A-3	100541034	43554 (0.04%)	43541 (0.04%)	13 (0%)

Table S3: Protein Identification Overview.

Sample	Total spectra	Matched spectra	Peptide	Identified protein
CQ10-P-1	72315	6188	4779	1094
CQ10-P-2	72416	6474	5139	1224
CQ10-P-3	71017	5366	4037	955
CQ10-A-1	63950	27819	21420	3409
CQ10-A-2	57493	1118	836	271
CQ10-A-3	61123	21348	15743	2733
Total	398314	68313	51954	9686

Table S4: Co-expressed protein in alfalfa and mice.

Protein ID	Gene ID	Uniprot Protein name	Gene name
A0A6B3IR79	K6R05_RS12585	Formate acetyltransferase	*PflB*
A0A1T4KN39	K6R05_RS10495	Major outer membrane lipoprotein Lpp (DEPs)	*lpp* (DEGs)
A0A349IFL6	K6R05_RS03700	26 kDa periplasmic immunogenic protein	*CBF16_15560*
A0A7X2SV25	K6R05_RS17645	Elongation factor Tu, EF-Tu	*tuf*
A0A1T4LCN6	K6R05_RS08555	Glyceraldehyde-3-phosphate dehydrogenase	*gapA*
A0A1T4SCA6	K6R05_RS17630	50S ribosomal protein L11	*rplK*

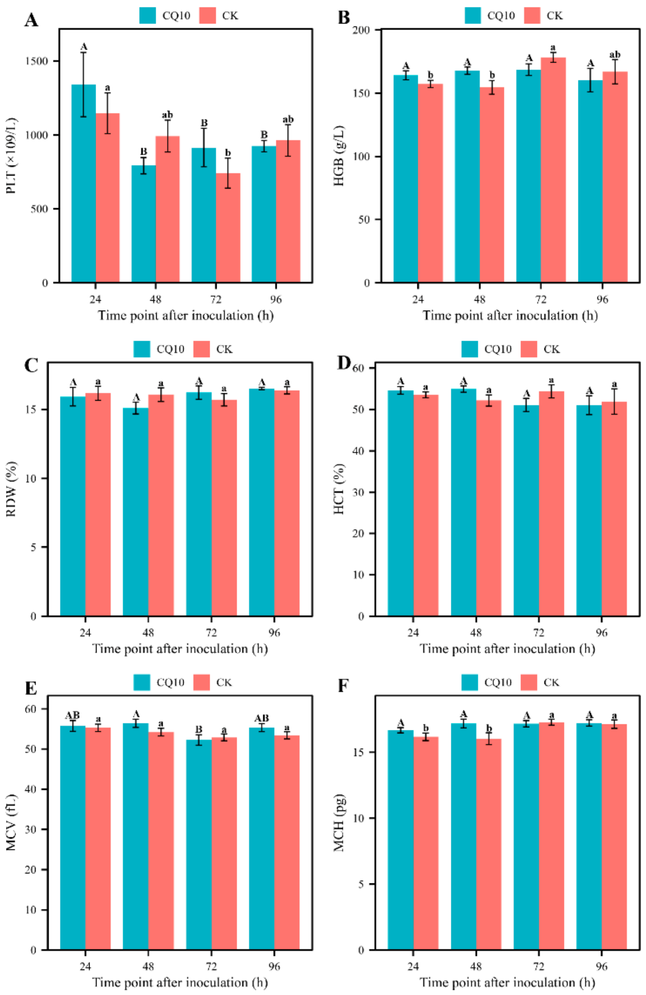

Figure S1: A, Platelet (PLT) counts. B, Hb hemoglobin (HGB) counts. C, The red blood cell distribution width-coefficient of variation (RDW). D, Hematocrit (HCT). E, Mean cell volume (MCV). F, mean corpuscular hemoglobin (MCH) concentration.

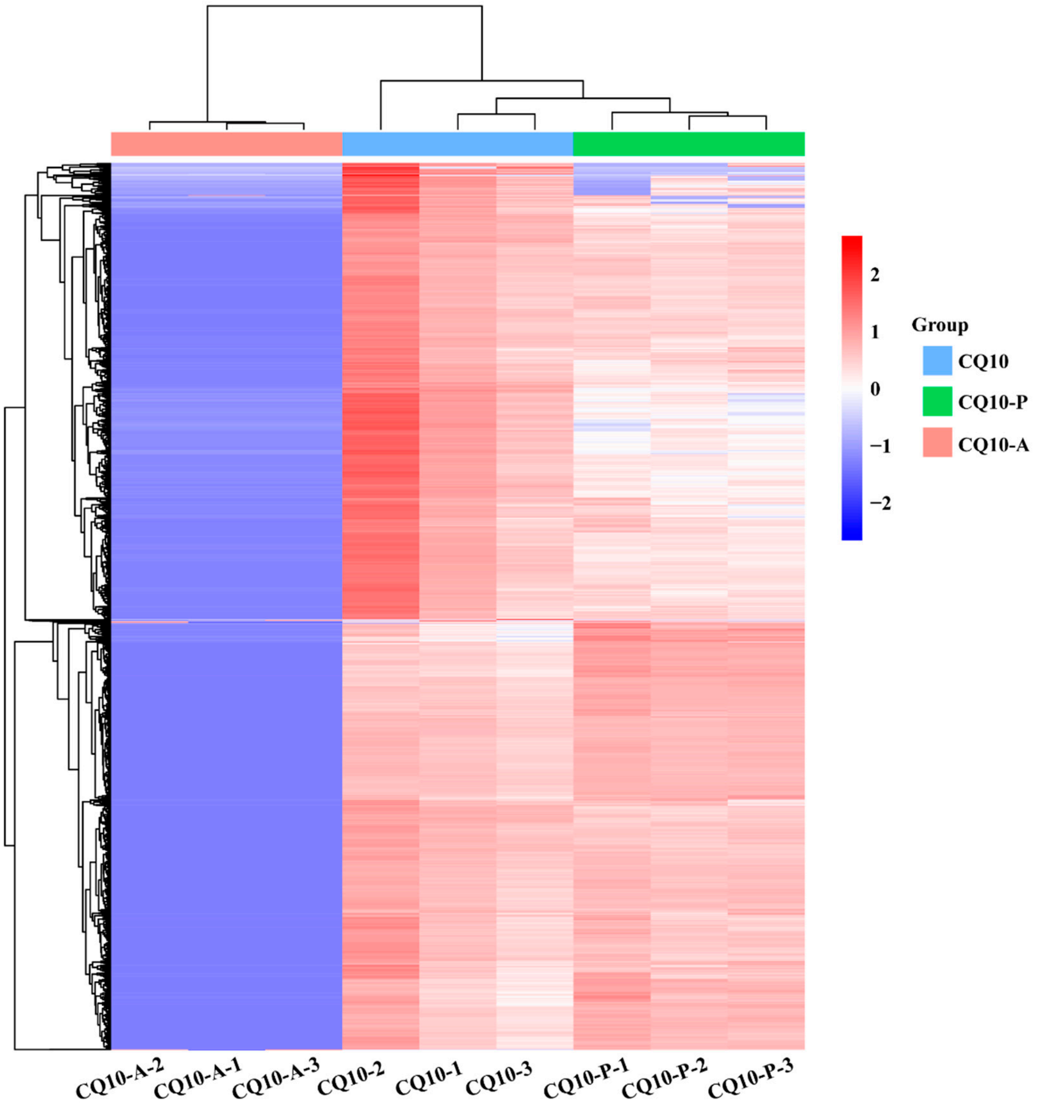

Figure S2: Clustering analysis by total DEGs. The horizontal coordinates refer to sample group, and the vertical coordinates refer to the expression of DEGs. Red for upregulation and blue for downregulation.

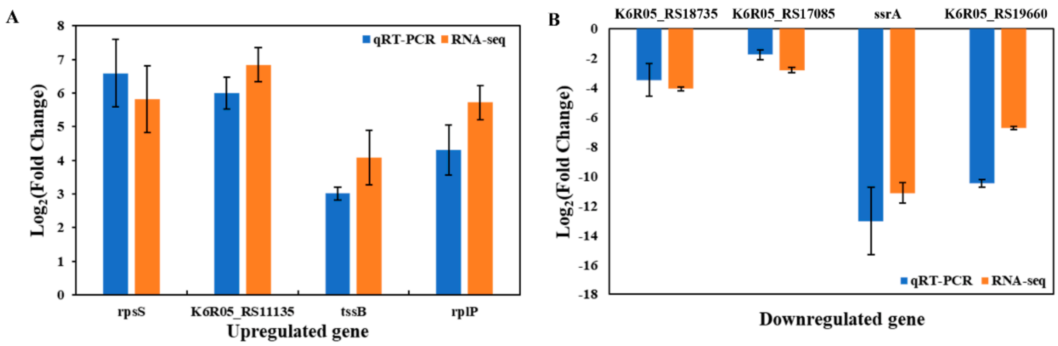

Figure S3: RTq-PCR verification of DEGs. A, RTq-PCR verification of upregulated gene. B, RTq-PCR verification of downregulated gene.

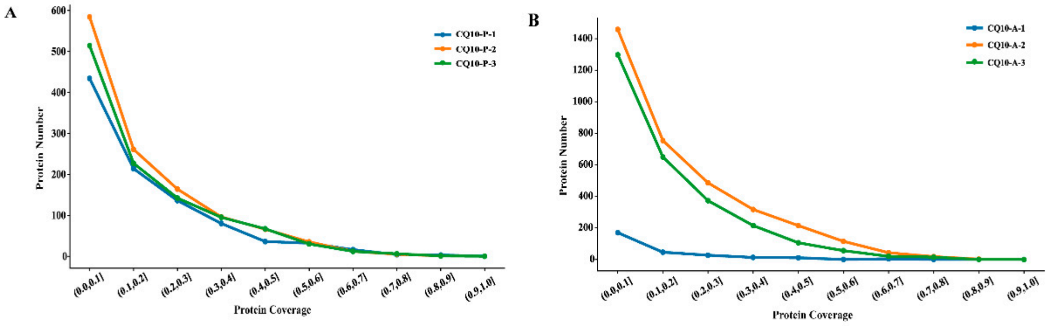

Figure S4: Evaluation results of proteomic quality control. A, The distribution of protein coverage of CQ10-P group. B, The distribution of protein coverage of CQ10-A group.

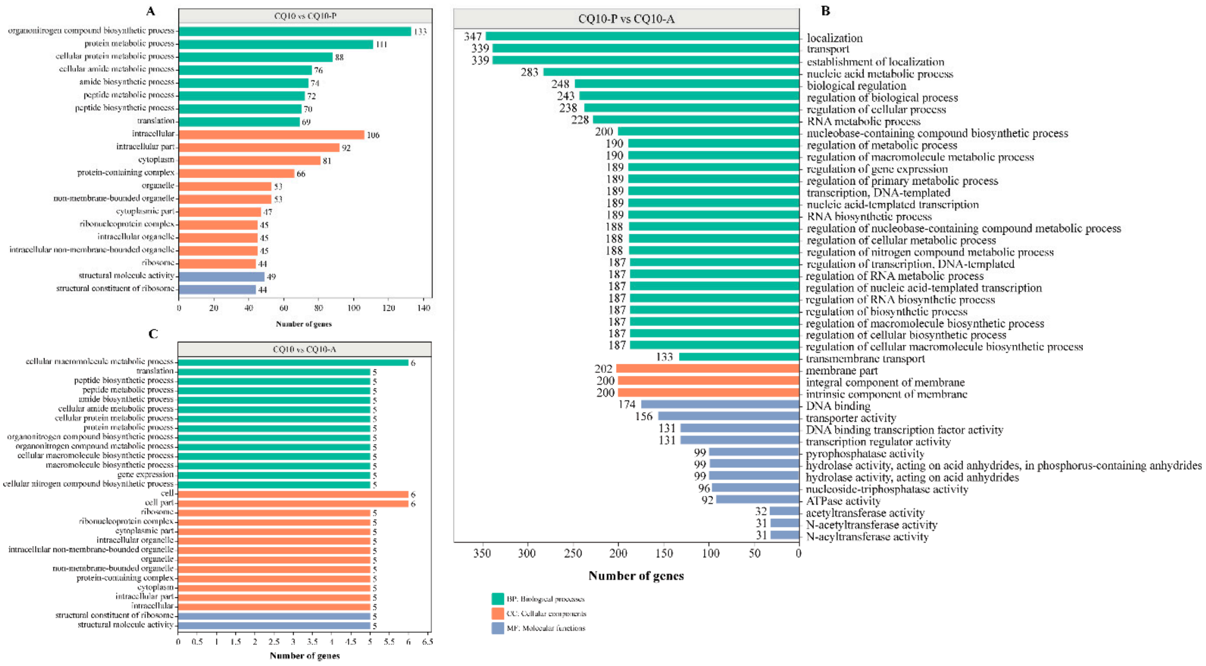

Figure S5: The GO annotated entries containing the largest DEGs. A, The top 20 GO annotated entries containing the largest DEGs in CQ10 group and CQ10-P group. B, The top 20 GO annotated entries containing the largest DEGs in CQ10-P group and CQ10-A group. C, The GO annotated entries containing the largest DEGs in CQ10 group and CQ10-A group.

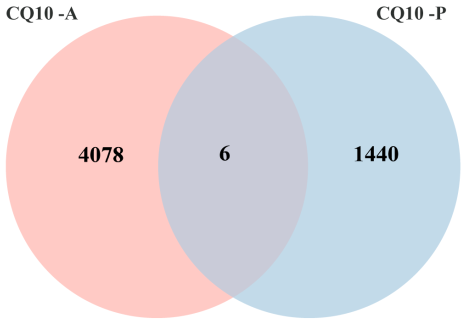

Figure S6: Venn analysis of number of DEPs.

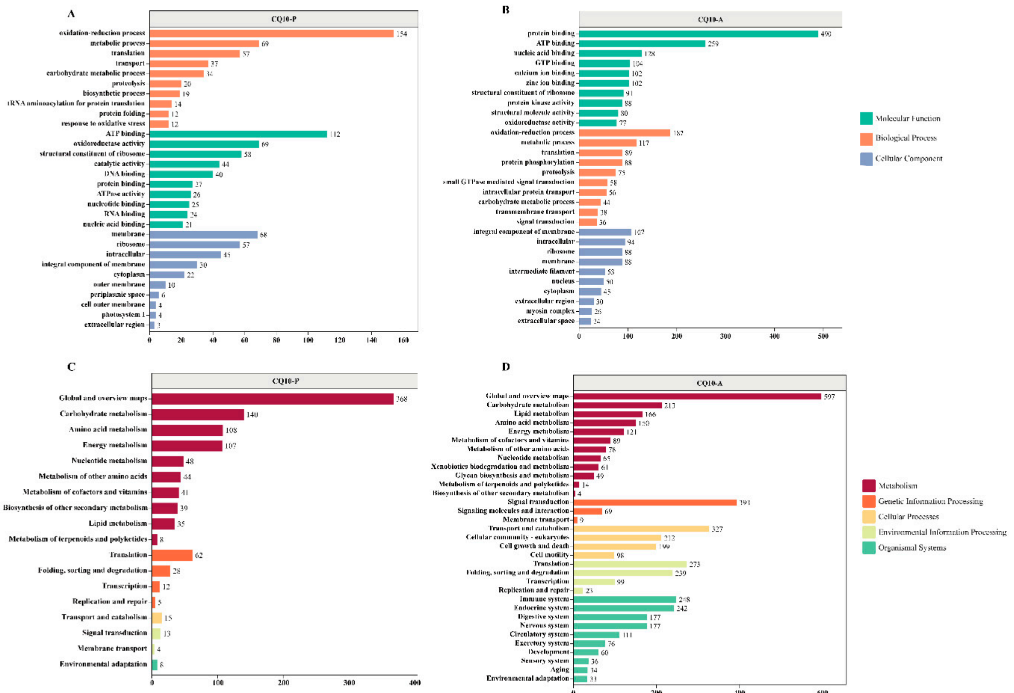

Figure S7: The GO annotated entries and KEGG pathways containing the largest DEPs. A, The top 30 GO annotated entries containing the largest DEPs in CQ10-P group. B, The 30 top GO annotated entries containing the largest DEPs in CQ10-A group. C, The KEGG pathways containing the largest DEPs in CQ10-P group. D, The KEGG pathways containing the largest DEPs in CQ10-A group.

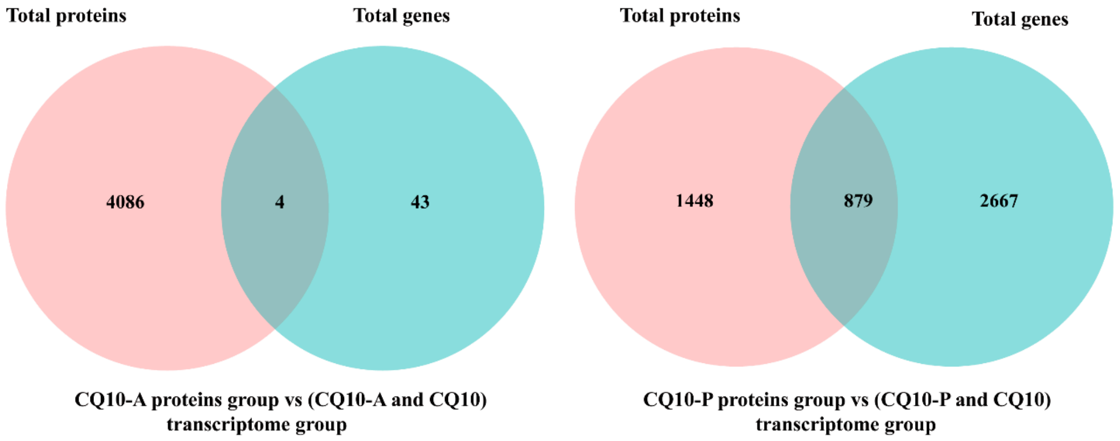

Figure S8: Joint Venn diagram analysis for total DEGs/DEPs.
